# Crystal packing in three related disaccharides: precursors to heparan sulfate oligosaccharides

**DOI:** 10.1107/S2056989015008518

**Published:** 2015-05-09

**Authors:** Graeme J. Gainsford, Ralf Schwörer, Peter C. Tyler, Olga V. Zubkova

**Affiliations:** a CallaghanInnovation, PO Box 31-310, Lower Hutt 5040, New Zealand; b Ferrier Research Institute, Victoria University of Wellington, PO Box 33 436, Petone, Lower Hutt 5046, New Zealand

**Keywords:** crystal structure, disaccharide, Alzheimer’s, synthesis, hydrogen bonding, C—H⋯π contacts

## Abstract

The three title compounds form part of a set of important precursor dissacharides which lead to novel therapeutics, in particular for Alzheimer’s disease. All three crystallize as poorly diffracting crystals with one independent mol­ecule in the asymmetric unit. Two of them are isostructural: 4-meth­oxy­phenyl 4-*O*-[6-*O*-acetyl-2-azido-3-*O*-benzyl-2-de­oxy-4-*O*-(9-fluor­en­yl­methyl­oxycarbon­yl)-α-d-gluco­pyranos­yl]-2-*O*-benzoyl-3-*O*-benzyl-6-*O*-chloro­acetyl-α-l-ido­pyran­oside, C_59_H_56_ClN_3_O_16_, (I), the *ido*-relative of a reported *gluco*-disaccharide [Gainsford *et al.*, 2013 ▸). *Acta Cryst.* C**69**, 679–682] and 4-meth­oxy­phenyl 4-*O*-[6-*O*-acetyl-2-azido-3-*O*-benzyl-2-de­oxy-4-*O*-(9-fluorenyl­methyl­oxycarbon­yl)-α-d-gluco­pyranos­yl]-2-*O*-benzoyl-3-*O*-benzyl-6-*O*-meth­oxy­acetyl-α-l-ido­pyran­oside, C_60_H_59_N_3_O_17_, (II). Both exhibit similar conformational disorder of pendant groups. The third compound 4-meth­oxy­phenyl 4-*O*-[6-*O*-acetyl-2-azido-3,4-di-*O*-benzyl-2-de­oxy-α-d-gluco­pyranos­yl]-2-*O*-benzoyl-3-*O*-benzyl-6-*O*-meth­oxy­oacetyl-β-d-gluco­pyran­oside, C_52_H_55_N_3_O_15_, (III), illustrates that a slightly larger set of weak inter­molecular inter­actions can result in a less disordered mol­ecular arrangement. The mol­ecules are bound by weak C—H⋯O(ether) hydrogen bonds in (I) and (II), augmented by C—H⋯π inter­actions in (III). The absolute configurations were determined, although at varying levels of significance from the limited observed data.

## Abstract 2

The structures of three disaccharide mol­ecules, precursors to novel therapeutics, as determined from weakly diffracting crystals are presented. The crystal packing depends mainly on weak C—H⋯O hydrogen-bond inter­actions, augmented by C—H⋯π contacts in the best-defined structure.

## Chemical context   

Heparan sulfate (HS) is a linear polysaccharide with a disaccharide repeating unit of d-glucosa­mine and l-iduronic or d-glucuronic acid, which can be *O*- or *N*-sulfated or *N*-acetyl­ated. HS is involved in the regulation of many important biological processes (Bishop *et al.*, 2007 ▸; Turnbull *et al.*, 2001 ▸). Synthetic HS-oligosaccharides with high potency as β-secretase (BACE1) inhibitors might have an application as novel therapeutics for Alzheimer’s disease (Schwörer *et al.*, 2013 ▸; Scholefield *et al.*, 2003 ▸).

In our recent paper (Schwörer *et al.*, 2013 ▸), we described the synthesis and inhibition data of a library of such oligosaccharides. At the centre of the synthetic methodology are highly orthogonally protected disaccharide building blocks, three of them being the subjects of this paper. The disaccharides can be converted into glycosyl donors by hydrolysis of the meth­oxy­phenyl glycoside and formation of the corresponding tri­chloro­acemidate; while the azide and the orthogonal ester protecting groups provide selective access to further functionalization later in the synthesis.

While pursuing precursor disaccharides with possible application in the treatment of Alzheimer’s disease, we have prepared some *ido*- and *gluco-*related crystals of the published *gluco*-derivative 4-meth­oxy­phenyl 4-*O*-[6-*O*-acetyl-2-azido-3-*O*-benzyl-2-de­oxy-4-*O*-(9-fluorenyl­methyl­oxycarbon­yl)-α-d-gluco­pyranos­yl]-2-*O*-benzoyl-3-*O*-benzyl-6-*O*-chloro­acetyl-β-d-gluco­pyran­oside, hereafter RSTE (Gainsford *et al.*, 2013 ▸). We have been intrigued that no unambiguous defining set of inter­molecular attractive inter­actions has been observed (Gainsford *et al.*, 2012 ▸) for these four structures and three other in-house examples.
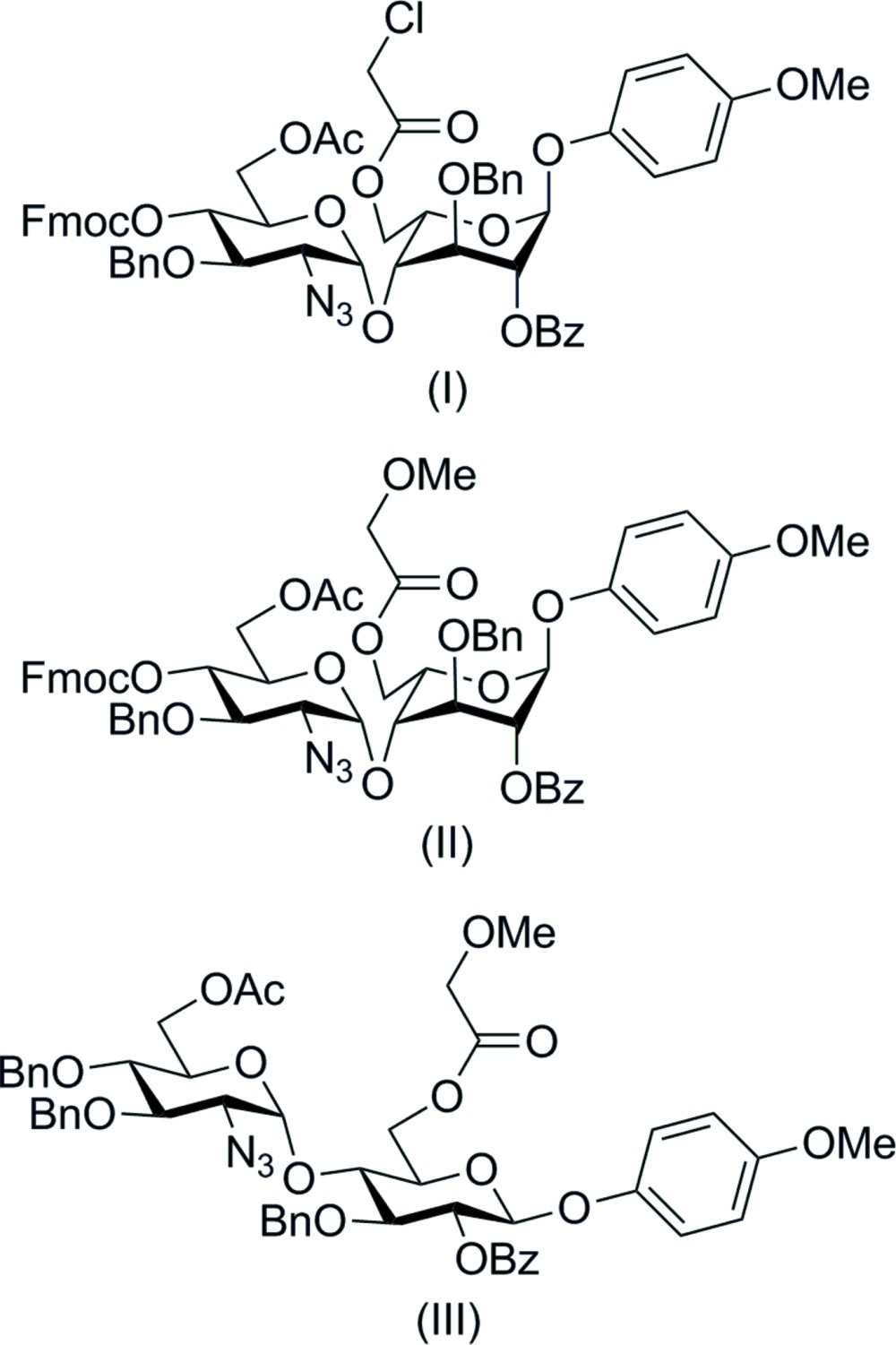


## Structural commentary   

**4-Meth­oxy­phenyl 4-** ***O*** **-[6-** ***O*** **-acetyl-2-azido-3-** ***O*** **-benzyl-2-de­oxy-4-** ***O*** **-(9-fluorenyl­methyl­oxycarbon­yl)-α-D-gluco­pyranos­yl]-2-** ***O*** **-benzoyl-3-** ***O*** **-benzyl-6-** ***O*** **-chloro­acetyl-α-l-ido­pyrano­side, (I) (hereafter OZTF)**

**Figure 1:**
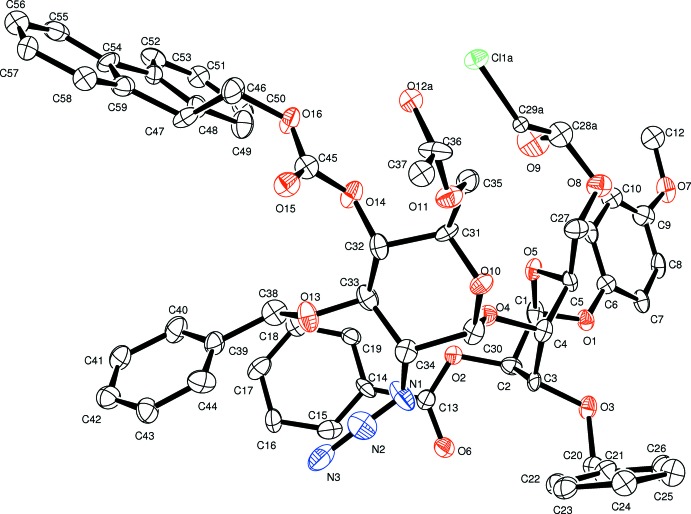
An *ORTEP-3* (Farrugia, 2012 ▸) view of (I) showing the asymmetric unit and labels with 20% probability ellipsoids. H atoms have been omitted for clarity. Only one (*A*) of the two disordered conformations for atoms C28, C29, O9 and Cl1, and C37, C37 and O12 (see text) are shown.

The asymmetric unit contains one independent mol­ecule of the title compound (Fig. 1 ▸) with the pyran­ose rings in chair conformations (Table 1 ▸). The determined absolute configuration confirmed the expected stereochemistry: C1(*S*), C2(*R*), C3(*S*), C4(*S*), C5(*S*), C30(*S*), C31(*R*), C32(*S*), C33(*R*), C34(*R*), C47(*R*). Conformational two-site disorder models were required for the pendant 6-*O*-chloro­acetyl and methyl of the 6-*O*-acetyl groups.

**(I4-Meth­oxy­phenyl 4-** ***O*** **-[6-** ***O*** **-acetyl-2-azido-3-** ***O*** **-benzyl-2-de­oxy-4-** ***O*** **-(9-fluorenyl­methyl­oxycarbon­yl)-α-D-gluco­pyranos­yl]-2-** ***O*** **-benzoyl-3-** ***O*** **-benzyl-6-** ***O*** **-meth­oxy­oacetyl-α-l-idopyran­oside, (II) (hereafter RNSB)**

**Figure 2:**
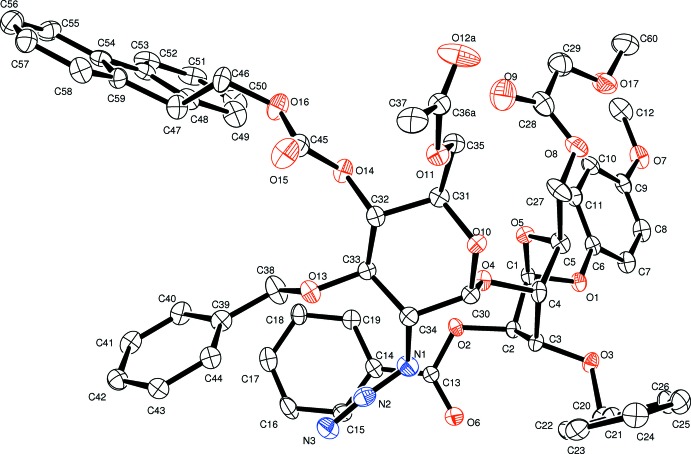
An *ORTEP-3* (Farrugia, 2012 ▸) view of (II) showing the asymmetric unit and labels with 30% probability ellipsoids. H atoms have been omitted for clarity. Only one (*A*) of the disordered conformations for atoms C36 and O12 (see text) are shown.

**Figure 3:**
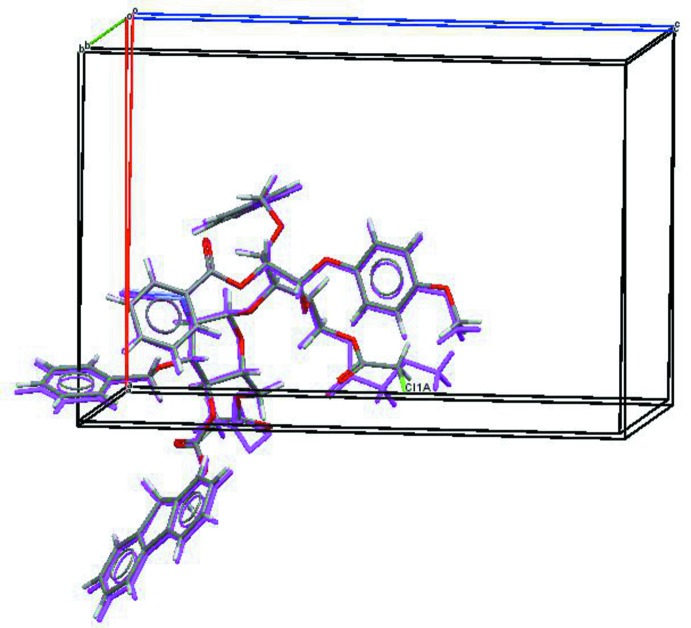
An overlap view (*Mercury;* Macrae *et al.* (2008 ▸) of the cell and asymmetric-unit atoms for the isostructural mol­ecules (I) (atom colours) and (II) (in purple). The Cl atom in (I) is labelled to highlight the different pendant groups.

This mol­ecule (Fig. 2 ▸) crystallized in an isostructural cell to (I), as shown in Fig. 3 ▸. A comparison of the mol­ecules of (I) and (II) shows that intra­molecular inter­actions seem to determine the near identical atomic configurations (see Figs. 1 ▸, 2 ▸ and 3 ▸). As might be expected, only one other weak packing inter­molecular inter­action is found.

**4-Meth­oxy­phenyl 4-** ***O*** **-[6-** ***O*** **-acetyl-2-azido-3,4-** ***O*** **-benzyl-2-de­oxy-α-d-gluco­pyranos­yl]-2-** ***O*** **-benzoyl-3-** ***O*** **-benzyl-6-** ***O*** **-meth­oxy­acetyl-β-d-gluco­pyran­oside, (III) (hereafter RSTN)**

**Figure 4:**
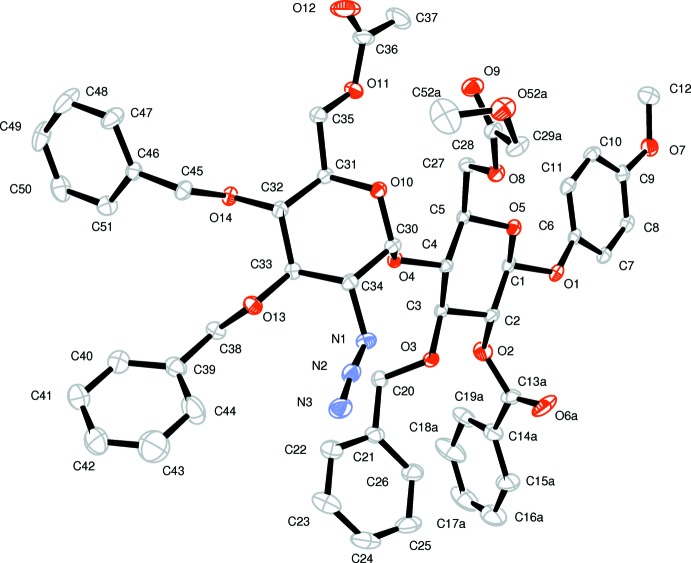
An *ORTEP-3* (Farrugia, 2012 ▸) view of (III) showing the asymmetric unit and labels with 30% probability ellipsoids. H atoms have been omitted for clarity. Only one (*A*) of the disordered conformations for atoms C13–C19 and O6, and C29, C52 and O52 (see text) are shown.

Compound (III) (Fig. 4 ▸) crystallizes with one independent mol­ecule in the asymmetric unit but with disorder on one of the terminal benz­yloxy groups and the 2-meth­oxy­acet­oxy methyl group, modelled by two-site disorder models. The absolute configuration was not ambiguously determined but is known from the synthetic chemistry.

The conformational data given in Tables 1 ▸ and 2 ▸ show the essential pyran­ose chair conformations have not been disturbed significantly in the title compounds.

## Supra­molecular features   

**Figure 5:**
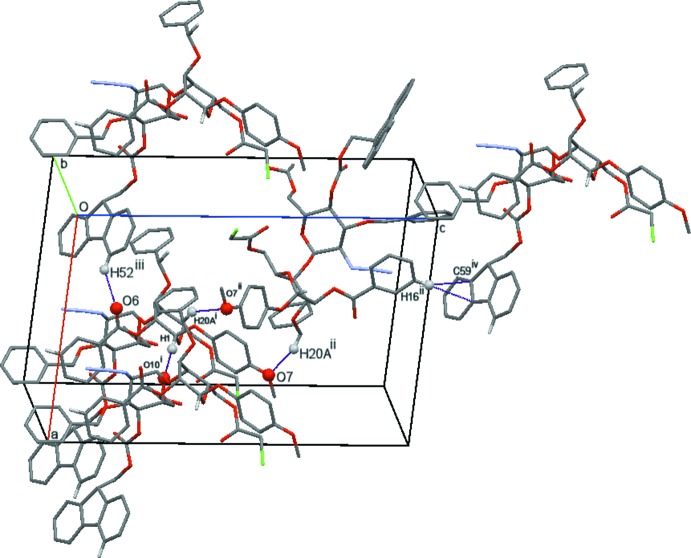
Cell-packing view (Macrae *et al.*, 2008 ▸) of (I) showing representative hydrogen-bonding inter­actions (see Table 3 ▸). The C—H⋯π inter­action is shown by atoms H16 and C59. [Symmetry codes: (i) *x*, *y* − 1, *z*; (ii) −*x* + 1, *y* − 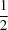, −*z* + 1; (iii) *x* − 1, *y*, *z*; (iv) *x* − 1, *y* − 1, *z* + 1.]

The crystal packing in (I) is provided by weak C—H⋯O(ether), C—H⋯O (carbon­yl) hydrogen bonds and one C—H⋯π inter­action (Table 3 ▸). These inter­actions form a three-dimensional network in which the base motifs are *C*(8), *C*(12) and *C*(20) (Bernstein *et al.*, 1995 ▸; Fig. 5 ▸). Given the unusual pseudo-dimeric nature of the hydrogen bonding in the gluco­pyran­oside crystal (Gainsford *et al.*, 2013 ▸) and the chloro­acet­oxy group disorder, it is not surprising that there is only one common C—H⋯O(carbon­yl) inter­action involving the C1—H1 atoms. In the isostructural compound (II), the same inter­actions are observed plus one additional methyl­ene-H⋯O(ether) (C29—H29⋯O12*A*) interaction (Table 4 ▸); this is only possible in (II) with the difference in composition of the two mol­ecules (the chloro­acetyl being replaced by the meth­oxy­acetyl group).

In (III), the five C—H⋯O(ether and ketone) inter­actions are augmented by five C—H⋯π inter­actions (Table 5 ▸). These inter­actions form stacks of twofold-related mol­ecules along the *b* axis in which 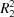
(18) and *C*(*n*) (*n* = 5,17) motifs (Bernstein *et al.*, 1995 ▸) are present.

## Database survey   

There are only a few reported 2-azido pyran­ose-based disaccharide structures in the Cambridge Structural Database (Version 5.36, with February 2015 update; Groom & Allen, 2014 ▸): our published gluco­pyran­oside (Gainsford *et al.*, 2013 ▸; BILJAJ), a manno­pyran­oside (Luger & Paulsen, 1981 ▸; BABHUH) and one ido­pyran­ose (Lee *et al.*, 2004 ▸; AQOGIW). We note another disaccharide gluco­pyran­ose (Abboud *et al.*, 1997 ▸; RAVNAD) for comparison. The conformational data given in Tables 1 ▸ and 2 ▸ show the pyran­ose essential chair conformations have not been disturbed significantly, although the ring with the bound azide seems to be closer to a ‘pure’ chair conformation by the θ criteria (Cremer & Pople, 1975 ▸).

## Synthesis and crystallization   

The title compounds were prepared as described in Schwörer *et al.* (2013 ▸). Crystals were obtained by vapour diffusion of petroleum ether into a solution of the title compounds in ethyl acetate (I) or toluene (II) and (III).

## Refinement   

Crystal data, data collection and structure refinement details are summarized in Table 6 ▸. Subject to variations noted below, the methyl H atoms were constrained to an ideal geometry (C—H = 0.98 Å) with *U*_iso_(H) = 1.5*U*_eq_(C), but were allowed to rotate freely about the adjacent C—C bonds. All other H atoms were placed in geometrically idealized positions and constrained to ride on their parent atoms with C—H distances of 0.95 (aromatic), 0.99 (methyl­ene) or 1.00 (tertiary) Å with *U*_iso_(H) = 1.2*U*_eq_(C) or 1.5*U*_eq_(C) (for methyl C) of their parent atom. Specific variations were:

(I) Data at resolution less than 1.12 Å was not significantly above the noise level and was excluded from the refinement. One other reflection (1,0,9) was OMITted as an outlier. Data analysis shows that there are many data in the resolution range 1.40–1.12 Å that are in poor agreement reflecting crystal quality.

There was conformational disorder in the chloro­acet­oxy (atoms C28, C29, O9 and Cl1) and the meth­oxy­carbon­yloxy (atoms C37, C37 and O12) groups which was modelled as two (*A* and *B*) groups. Because of proximity, and poor data quality, these atoms were unable to be refined with anisotropic thermal parameters. It proved advisable to add additional restraints to retain known geometries based on published structures for these groups. So (*SHELXL* DFIX) C28–C29 pairs were held to 1.50 (3) Å; C28—O9 to 1.20 Å and same-distance constraints (SADI, 0.02) were applied to C29–Cl1, C36–C37 and C36–O11. Thermal parameters were also linked using SIMU for ring atoms C6–C11 and atom pairs C53 and C54, O12*A* and O12*B*, C37*A* and C37*B*, and C36*A* and C36*B*. Finally, rings C6–C11 and C14–C19 were constrained to hexa­gonal geometry with C—C = 1.390 Å. Final *A*:*B* occupancies for the chloro­acet­oxy group were 0.509 (17):0.491 (17) and for the meth­oxy­carbon­yloxy, 0.44 (4):0.56 (4).

(II) Data at resolution less than 0.81 Å was not significantly above the noise level and was excluded from the refinement. Two reflections (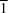
7,1,7; 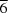,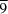,5) were OMITted as clear outlier data. There was two-site conformational disorder for the meth­oxy­lacetyl atoms C36 and O12 (labelled *A* and *B*, respectively). Atoms C13, C33, C34, C30, C361 and C36*B* were restrained to isotropic-like behaviour (using ISOR) and the two-model disordered atoms (O12*A*, O12*B*; C36*A*, C36*B*) were given the same anisotropic thermal parameters. Distance constraints (SADI, 0.3) were applied to the C36*A*—O12*A* and C36*B*—O12*B* bonds. Final *A*:*B* occupancies for the meth­oxy­acetyl atoms were 0.797 (16):0.203 (16).

(III) One reflection was removed as an outlier as well as nine low angle reflections affected by the beamstop (*F*_o_<<*F*_c_). The mol­ecule showed two major orientations for the benzyl group (atoms C13–C19) refined by two refining set occupancies [*A*:*B* 0.793 (6):0.207 (6)] coupled with equivalent *U* values (SIMU for each ring set) and with each ring restrained to a regular hexa­gon (C—C 1.39 Å). In a similar manner, two orientations of atoms C29, O52 and C52 were refined as two conformations: final *A*:*B* ratio 0.687 (8):0.313 (8).

## Supplementary Material

Crystal structure: contains datablock(s) global, OZTF, RNSB, RSTN. DOI: 10.1107/S2056989015008518/sj5456sup1.cif

Structure factors: contains datablock(s) OZTF. DOI: 10.1107/S2056989015008518/sj5456OZTFsup2.hkl

Structure factors: contains datablock(s) RNSB. DOI: 10.1107/S2056989015008518/sj5456RNSBsup3.hkl

Structure factors: contains datablock(s) RSTN. DOI: 10.1107/S2056989015008518/sj5456RSTNsup4.hkl

Supporting information file. DOI: 10.1107/S2056989015008518/sj5456RSTNsup5.cml

CCDC references: 1062536, 1062535, 1062534

Additional supporting information:  crystallographic information; 3D view; checkCIF report

## Figures and Tables

**Table 1:** Conformational parameters (Å, °) (Cremer & Pople, 1975 ▸) for *iodo-*pyran­ose rings Head_D and Foot_D represent the distance from the four-atom ‘seat’ plane.

Compound	ring	*Q*	Θ	φ	Head_D	Foot_D
(I)	C1–C5,O5	0.54 (3)	161 (3)	150 (8)	0.685 (17)	−0.47 (2)
(II)	C1–C5,O5	0.532 (8)	161.8 (9)	140 (3)	0.669 (4)	−0.478 (7)
(I)	C30–C34,O10	0.57 (3)	4(3)	241 (38)	0.67 (3)	−0.68 (3)
(II)	C30–C34,O10	0.564 (8)	1.2 (8)	10 (24)	0.646 (5)	−0.651 (8)
ADOGIW^*a*^		0.562	5.5	329	0.656 (4)	−0.622 (7)

Notes: (*a*) AQOGIW (Lee *et al.*, 2004 ▸).

**Table 2:** Conformational parameters (Å, °) (Cremer & Pople, 1975 ▸) for *gluco*-pyran­ose rings^*a*^ Head_D and Foot_D represent the distance from the four-atom ‘seat’ plane.

Compound	ring	*Q*	Θ	φ	Head_D	Foot_D
(III)	C1–C5,O5	0.613 (3)	7.3 (3)	323 (2)	0.714 (2)	−0.662 (3)
RSTE-1^*a*^		0.588 (8)	11.8 (8)	293 (4)	0.748 (8)	−0.586 (8)
RSTE-2^*a*^		0.594 (8)	14.6 (8)	288 (3)	0.768 (8)	−0.566 (8)
(III)	C30–C34,O10	0.591 (3)	1.7 (3)	150 (6)	0.716 (3)	−0.639 (2)
RSTE-1^*b*^		0.582 (8)	0.0 (8)	202 (41)	0.666 (8)	−0.692 (8)
RSTE-1^*b*^		0.561 (3)	3.9 (9)	116 (13)	0.675 (8)	−0.648 (8)
RAVNAD-1		0.597 (3)	7.5 (3)	89 (2)	0.727 (4)	−0.652 (4)
RAVNAD-2		0.577 (3)	13.8 (3)	340.8 (13)	0.713 (4)	−0.555 (5)

Notes: (*a*) RSTE mol­ecules 1 and 2 (Gainsford *et al.*, 2013 ▸); (*b*) RAVNAD (Abboud *et al.*, 1997 ▸).

**Table 3:** Hydrogen-bond geometry (Å, °) for OZTF[Chem scheme1] *Cg*9 is the centroid of the C54–C59 ring.

*D*—H⋯*A*	*D*—H	H⋯*A*	*D*⋯*A*	*D*—H⋯*A*
C1—H1⋯O10^i^	1.00	2.53	3.51 (3)	169
C20—H20*A*⋯O7^ii^	0.99	2.57	3.44 (3)	146
C52—H52⋯O6^iii^	0.95	2.46	3.26 (3)	142
C16—H16⋯*Cg*9^iv^	0.95	2.65	3.520 (12)	152

Symmetry codes: (i) 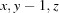; (ii) 
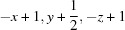
; (iii) 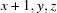; (iv) 
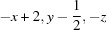
.

**Table 4:** Hydrogen-bond geometry (Å, °) for RNSB[Chem scheme1]

*D*—H⋯*A*	*D*—H	H⋯*A*	*D*⋯*A*	*D*—H⋯*A*
C1—H1⋯O10^i^	1.00	2.43	3.395 (9)	161
C3—H3⋯O6	1.00	2.63	3.114 (10)	110
C11—H11⋯O5	0.95	2.37	2.987 (10)	122
C20—H20*A*⋯O7^ii^	0.99	2.50	3.370 (11)	147
C29—H29*B*⋯O12*A* ^iii^	0.99	2.53	3.503 (14)	168
C29—H29*B*⋯O12*B* ^iii^	0.99	2.60	3.55 (4)	159

Symmetry codes: (i) 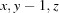; (ii) 
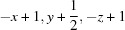
; (iii) 
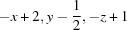
.

**Table 5:** Hydrogen-bond geometry (Å, °) for RSTN[Chem scheme1] *Cg*3, *Cg*5 and *Cg*6 are the centroids of the C6–C11, C21–C26 and C39–C44 phenyl rings, respectively.

*D*—H⋯*A*	*D*—H	H⋯*A*	*D*⋯*A*	*D*—H⋯*A*
C5—H5⋯O1^i^	1.00	2.46	3.439 (4)	168
C27—H27*B*⋯O11	0.99	2.55	3.499 (4)	161
C45—H45*A*⋯O12^ii^	0.99	2.51	3.488 (5)	168
C47—H47⋯O52*A* ^iii^	0.95	2.59	3.269 (4)	128
C48—H48⋯O9^iii^	0.95	2.65	3.569 (6)	164
C3—H3⋯*Cg*3^i^	1.00	2.96	3.915 (3)	161
C4—H4⋯*Cg*3^iv^	1.00	2.96	3.920 (3)	161
C12—H12*B*⋯*Cg*5^i^	0.98	2.71	3.563 (3)	145
C16*A*—H16*A*⋯*Cg*6^v^	0.95	2.88	3.713 (3)	147
C25—H25⋯*Cg*5^v^	0.95	2.94	3.717 (4)	140

Symmetry codes: (i) 
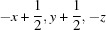
; (ii) 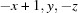; (iii) 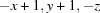; (iv) 
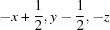
; (v) 
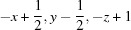
.

**Table 6:** Experimental details

	OZTF	RNSB	RSTN
Crystal data			
Chemical formula	C_59_H_56_ClN_3_O_16_	C_60_H_59_N_3_O_17_	C_52_H_55_N_3_O_15_
*M*_r_	1098.51	1094.10	961.99
Crystal system, space group	Monoclinic, *P*2_1_	Monoclinic, *P*2_1_	Monoclinic, *C*2
Temperature (K)	123	120	118
*a*, *b*, *c* (Å)	14.8343 (11), 8.4771 (6), 21.8112 (17)	14.8595 (17), 8.3873 (6), 22.0138 (18)	38.3346 (13), 8.0744 (3), 16.1659 (6)
β (°)	91.780 (7)	90.939 (10)	91.222 (2)
*V* (Å^3^)	2741.5 (4)	2743.2 (4)	5002.7 (3)
*Z*	2	2	4
Radiation type	Cu *K*α	Cu *K*α	Mo *K*α
μ (mm^−1^)	1.24	0.81	0.09
Crystal size (mm)	0.6 × 0.05 × 0.02	0.36 × 0.06 × 0.01	0.75 × 0.32 × 0.30

Data collection			
Diffractometer	Rigaku Spider	Agilent SuperNova (Dual, Cu at zero, Atlas)	Bruker APEXII CCD
Absorption correction	Multi-scan (*ABSCOR*; Higashi, 1995 ▸)	Gaussian (*CrysAlis PRO*; Agilent, 2013 ▸)	Multi-scan (Blessing, 1995 ▸)
*T*_min_, *T*_max_	0.68, 1.0	1.080, 1.638	0.645, 0.745
No. of measured, independent and observed [*I* > 2σ(*I*)] reflections	19701, 3962, 2294	17226, 7922, 4977	51621, 9796, 9128
*R*_int_	0.101	0.101	0.035
θ_max_ (°)	43.5	72.1	26.1
(sin θ/λ)_max_ (Å^−1^)	0.446	0.617	0.619

Refinement			
*R*[*F* ^2^ > 2σ(*F* ^2^)], *wR*(*F* ^2^), *S*	0.088, 0.280, 1.09	0.083, 0.201, 1.04	0.048, 0.129, 1.08
No. of reflections	3962	7922	9796
No. of parameters	666	730	662
No. of restraints	55	38	43
H-atom treatment	H-atom parameters constrained	H-atom parameters constrained	H-atom parameters constrained
Δρ_max_, Δρ_min_ (e Å^−3^)	0.29, −0.25	0.36, −0.32	0.33, −0.45
Absolute structure	Parsons & Flack (2004 ▸), 1721 Friedel pairs	Flack *x* determined using 810 quotients [(*I* ^+^)−(*I* ^−^)]/[(*I* ^+^)+(*I* ^−^)] (Parsons & Flack, 2004 ▸)	Flack *x* determined using 3878 quotients [(*I* ^+^)−(*I* ^−^)]/[(*I* ^+^)+(*I* ^−^)] (Parsons & Flack, 2004 ▸)
Absolute structure parameter	0.01 (8)	−0.3 (4)	0.0 (2)

Computer programs: *CrystalClear* (Rigaku, 2005 ▸), *FSProcess* (Rigaku, 1998 ▸), *CrysAlis PRO* (Agilent, 2013 ▸), *APEX2* and *SAINT* (Bruker, 2005 ▸), *SHELX-D* and *SHELXS97* (Sheldrick, 2008 ▸), *SHELXL2012* (Sheldrick, 2015 ▸), *ORTEP-3* in *WinGX* (Farrugia, 2012 ▸), *Mercury* (Macrae *et al.*, 2008 ▸) and *PLATON* (Spek, 2009 ▸).

## supplementary crystallographic information

### (OZTF) 4-Methoxyphenyl 4-O-[6-O-acetyl-2-azido-3-O-benzyl-2-deoxy-4-O-(9-fluorenylmethyloxycarbonyl)-α-D-glucopyranosyl]-2-O-benzoyl-3-O-benzyl-6-O-chloroacetyl-α-L-iodopyranoside Crystal data

**Table:**

C_59_H_56_ClN_3_O_16_	*Z* = 2
*M**_r_* = 1098.51	*F*(000) = 1152
Monoclinic, *P*2_1_	*D*_x_ = 1.331 Mg m^−^^3^
Hall symbol: P 2yb	Cu *K*α radiation, λ = 1.54178 Å
*a* = 14.8343 (11) Å	µ = 1.24 mm^−^^1^
*b* = 8.4771 (6) Å	*T* = 123 K
*c* = 21.8112 (17) Å	Needle, colourless
β = 91.780 (7)°	0.6 × 0.05 × 0.02 mm
*V* = 2741.5 (4) Å^3^	

### (OZTF) 4-Methoxyphenyl 4-O-[6-O-acetyl-2-azido-3-O-benzyl-2-deoxy-4-O-(9-fluorenylmethyloxycarbonyl)-α-D-glucopyranosyl]-2-O-benzoyl-3-O-benzyl-6-O-chloroacetyl-α-L-iodopyranoside Data collection

**Table:**

Rigaku Spider diffractometer	3962 independent reflections
Radiation source: Rigaku MM007 rotating anode	2294 reflections with *I* > 2σ(*I*)
Rigaku VariMax-HF Confocal Optical System monochromator	*R*_int_ = 0.101
Detector resolution: 10 pixels mm^-1^	θ_max_ = 43.5°, θ_min_ = 6.6°
ω–scans	*h* = −13→13
Absorption correction: multi-scan (*ABSCOR*; Higashi, 1995)	*k* = −7→7
*T*_min_ = 0.68, *T*_max_ = 1.0	*l* = −19→19
19701 measured reflections	

### (OZTF) 4-Methoxyphenyl 4-O-[6-O-acetyl-2-azido-3-O-benzyl-2-deoxy-4-O-(9-fluorenylmethyloxycarbonyl)-α-D-glucopyranosyl]-2-O-benzoyl-3-O-benzyl-6-O-chloroacetyl-α-L-iodopyranoside Refinement

**Table:**

Refinement on *F*^2^	Hydrogen site location: inferred from neighbouring sites
Least-squares matrix: full	H-atom parameters constrained
*R*[*F*^2^ > 2σ(*F*^2^)] = 0.088	*w* = 1/[σ^2^(*F*_o_^2^) + (0.1736*P*)^2^] where *P* = (*F*_o_^2^ + 2*F*_c_^2^)/3
*wR*(*F*^2^) = 0.280	(Δ/σ)_max_ < 0.001
*S* = 1.09	Δρ_max_ = 0.29 e Å^−^^3^
3962 reflections	Δρ_min_ = −0.25 e Å^−^^3^
666 parameters	Extinction correction: *SHELXL2012* (Sheldrick, 2015), Fc^*^=kFc[1+0.001xFc^2^λ^3^/sin(2θ)]^-1/4^
55 restraints	Extinction coefficient: 0.0110 (13)
Primary atom site location: structure-invariant direct methods	Absolute structure: Parsons & Flack (2004), 1721 Friedel pairs
Secondary atom site location: difference Fourier map	Absolute structure parameter: 0.01 (8)

### (OZTF) 4-Methoxyphenyl 4-O-[6-O-acetyl-2-azido-3-O-benzyl-2-deoxy-4-O-(9-fluorenylmethyloxycarbonyl)-α-D-glucopyranosyl]-2-O-benzoyl-3-O-benzyl-6-O-chloroacetyl-α-L-iodopyranoside Special details

**Table:**

Geometry. All e.s.d.'s (except the e.s.d. in the dihedral angle between two l.s. planes) are estimated using the full covariance matrix. The cell e.s.d.'s are taken into account individually in the estimation of e.s.d.'s in distances, angles and torsion angles; correlations between e.s.d.'s in cell parameters are only used when they are defined by crystal symmetry. An approximate (isotropic) treatment of cell e.s.d.'s is used for estimating e.s.d.'s involving l.s. planes.

### (OZTF) 4-Methoxyphenyl 4-O-[6-O-acetyl-2-azido-3-O-benzyl-2-deoxy-4-O-(9-fluorenylmethyloxycarbonyl)-α-D-glucopyranosyl]-2-O-benzoyl-3-O-benzyl-6-O-chloroacetyl-α-L-iodopyranoside Fractional atomic coordinates and isotropic or equivalent isotropic displacement parameters (Å^2^)

**Table:**

	*x*	*y*	*z*	*U*_iso_*/*U*_eq_	Occ. (<1)
O1	0.6023 (10)	0.4612 (18)	0.4054 (7)	0.087 (4)	
O2	0.6713 (9)	0.5629 (17)	0.2568 (9)	0.083 (4)	
O3	0.4899 (10)	0.7299 (17)	0.3450 (6)	0.089 (4)	
O4	0.7060 (9)	0.898 (2)	0.3050 (7)	0.093 (5)	
O5	0.7223 (10)	0.627 (2)	0.3832 (7)	0.092 (5)	
O6	0.5503 (13)	0.546 (2)	0.1899 (6)	0.106 (5)	
O7	0.7099 (13)	0.057 (2)	0.5924 (7)	0.120 (6)	
O8	0.7842 (13)	0.789 (2)	0.4871 (8)	0.118 (6)	
O10	0.7549 (11)	1.159 (2)	0.3127 (7)	0.093 (5)	
O11	0.8860 (10)	1.394 (2)	0.3282 (8)	0.110 (5)	
O13	0.8501 (13)	1.017 (2)	0.1475 (8)	0.129 (6)	
O14	0.9718 (12)	1.0557 (19)	0.2549 (8)	0.115 (5)	
O15	1.0271 (10)	1.266 (2)	0.2053 (8)	0.113 (5)	
O16	1.1160 (11)	1.068 (2)	0.2435 (7)	0.113 (5)	
N1	0.669 (2)	0.923 (4)	0.1826 (14)	0.150 (11)	
N2	0.662 (2)	0.933 (4)	0.129 (2)	0.156 (11)	
N3	0.6536 (18)	0.919 (5)	0.0750 (18)	0.169 (13)	
C1	0.660 (2)	0.515 (3)	0.3614 (13)	0.093 (8)	
H1	0.6954	0.4221	0.3479	0.112*	
C2	0.6048 (17)	0.571 (3)	0.3053 (12)	0.089 (7)	
H2	0.5532	0.4983	0.2962	0.107*	
C3	0.5723 (15)	0.735 (3)	0.3119 (10)	0.080 (7)	
H3	0.5593	0.7804	0.2702	0.095*	
C4	0.6363 (16)	0.845 (3)	0.3470 (12)	0.082 (7)	
H4	0.6014	0.9389	0.3602	0.098*	
C5	0.6779 (17)	0.768 (4)	0.4036 (10)	0.089 (7)	
H5	0.6296	0.7389	0.4326	0.107*	
C6	0.6354 (13)	0.3663 (17)	0.4527 (6)	0.093 (7)	
C7	0.5739 (10)	0.2666 (19)	0.4799 (7)	0.088 (7)	
H7	0.5124	0.2676	0.4664	0.106*	
C8	0.6023 (10)	0.1655 (18)	0.5267 (7)	0.103 (7)	
H8	0.5603	0.0974	0.5453	0.123*	
C9	0.6923 (10)	0.1640 (16)	0.5465 (6)	0.115 (9)	
C10	0.7539 (9)	0.2637 (18)	0.5193 (7)	0.117 (9)	
H10	0.8154	0.2627	0.5328	0.140*	
C11	0.7254 (12)	0.3648 (18)	0.4724 (7)	0.096 (7)	
H11	0.7675	0.4330	0.4539	0.115*	
C12	0.8015 (15)	0.047 (3)	0.6153 (9)	0.155 (11)*	
H12A	0.8069	−0.0373	0.6459	0.233*	
H12B	0.8190	0.1478	0.6342	0.233*	
H12C	0.8412	0.0243	0.5813	0.233*	
C13	0.633 (2)	0.547 (3)	0.2014 (15)	0.105 (8)	
C14	0.6979 (9)	0.5065 (12)	0.1521 (6)	0.099 (8)	
C15	0.6717 (10)	0.501 (2)	0.0904 (6)	0.118 (9)	
H15	0.6105	0.5200	0.0783	0.141*	
C16	0.7349 (12)	0.469 (2)	0.0464 (6)	0.130 (11)	
H16	0.7170	0.4654	0.0042	0.157*	
C17	0.8244 (11)	0.4416 (17)	0.0641 (7)	0.124 (10)	
H17	0.8676	0.4194	0.0340	0.149*	
C18	0.8506 (10)	0.4467 (19)	0.1258 (8)	0.099 (8)	
H18	0.9117	0.4281	0.1379	0.119*	
C19	0.7874 (9)	0.4792 (18)	0.1698 (7)	0.096 (8)	
H19	0.8053	0.4827	0.2120	0.115*	
C20	0.4107 (12)	0.797 (3)	0.3127 (9)	0.096 (8)	
H20A	0.3555	0.7523	0.3303	0.116*	
H20B	0.4115	0.7657	0.2690	0.116*	
C21	0.4065 (16)	0.973 (3)	0.3166 (17)	0.090 (8)	
C22	0.4360 (15)	1.054 (5)	0.2668 (12)	0.097 (8)	
H22	0.4551	0.9979	0.2318	0.117*	
C23	0.4383 (13)	1.221 (5)	0.2671 (12)	0.108 (9)	
H23	0.4629	1.2782	0.2341	0.130*	
C24	0.4033 (18)	1.298 (4)	0.3172 (15)	0.120 (9)*	
H24	0.4005	1.4103	0.3178	0.144*	
C25	0.3723 (16)	1.211 (4)	0.3667 (12)	0.105 (8)	
H25	0.3515	1.2648	0.4018	0.126*	
C26	0.3716 (18)	1.052 (5)	0.3650 (14)	0.118 (10)	
H26	0.3467	0.9947	0.3978	0.142*	
C27	0.7463 (17)	0.877 (3)	0.4356 (13)	0.104 (8)	
H27A	0.7941	0.9072	0.4071	0.125*	
H27B	0.7163	0.9741	0.4498	0.125*	
C30	0.699 (2)	1.057 (4)	0.2811 (12)	0.099 (8)	
H30	0.6350	1.0941	0.2828	0.119*	
C31	0.8492 (13)	1.119 (3)	0.3138 (12)	0.088 (7)	
H31	0.8577	1.0116	0.3323	0.106*	
C32	0.8812 (17)	1.117 (3)	0.2484 (11)	0.092 (7)	
H32	0.8801	1.2247	0.2297	0.110*	
C33	0.824 (2)	0.999 (3)	0.2106 (12)	0.106 (8)	
H33	0.8344	0.8886	0.2252	0.127*	
C34	0.727 (2)	1.042 (3)	0.2128 (12)	0.103 (8)	
H34	0.7168	1.1461	0.1918	0.124*	
C35	0.8947 (16)	1.241 (4)	0.3555 (12)	0.102 (8)	
H35A	0.8663	1.2412	0.3960	0.123*	
H35B	0.9593	1.2150	0.3618	0.123*	
C38	0.887 (2)	0.876 (5)	0.1199 (14)	0.147 (11)*	
H38A	0.8466	0.7845	0.1273	0.177*	
H38B	0.9470	0.8521	0.1382	0.177*	
C39	0.893 (2)	0.906 (5)	0.0497 (12)	0.107 (9)	
C40	0.944 (2)	0.799 (4)	0.022 (2)	0.136 (11)	
H40	0.9743	0.7175	0.0440	0.164*	
C41	0.9504 (19)	0.813 (5)	−0.045 (2)	0.125 (10)	
H41	0.9842	0.7390	−0.0672	0.150*	
C42	0.907 (2)	0.934 (6)	−0.0734 (17)	0.130 (10)	
H42	0.9125	0.9448	−0.1165	0.157*	
C43	0.857 (2)	1.039 (4)	−0.0438 (19)	0.118 (9)	
H43	0.8269	1.1210	−0.0657	0.141*	
C44	0.8507 (19)	1.027 (4)	0.020 (2)	0.123 (10)	
H44	0.8169	1.1017	0.0419	0.148*	
C45	1.041 (3)	1.146 (5)	0.2311 (14)	0.111 (9)*	
C46	1.1930 (18)	1.125 (4)	0.2112 (13)	0.123 (9)	
H46A	1.2477	1.1201	0.2383	0.148*	
H46B	1.1832	1.2354	0.1984	0.148*	
C47	1.2043 (15)	1.024 (4)	0.1575 (14)	0.106 (8)	
H47	1.1497	1.0340	0.1297	0.127*	
C48	1.222 (2)	0.848 (4)	0.1713 (12)	0.103 (8)	
C49	1.167 (2)	0.744 (5)	0.2026 (13)	0.129 (10)	
H49	1.1094	0.7763	0.2166	0.155*	
C50	1.199 (2)	0.588 (4)	0.2126 (10)	0.111 (8)	
H50	1.1633	0.5135	0.2336	0.133*	
C51	1.285 (2)	0.542 (4)	0.1916 (12)	0.117 (9)*	
H51	1.3042	0.4362	0.1984	0.140*	
C52	1.3403 (18)	0.643 (4)	0.1616 (12)	0.108 (9)	
H52	1.3986	0.6136	0.1490	0.130*	
C53	1.306 (2)	0.791 (4)	0.1513 (11)	0.094 (8)	
C54	1.3472 (19)	0.934 (5)	0.1211 (12)	0.109 (9)	
C55	1.428 (2)	0.953 (4)	0.0919 (11)	0.120 (10)	
H55	1.4677	0.8650	0.0893	0.144*	
C56	1.453 (2)	1.098 (4)	0.0658 (13)	0.115 (9)*	
H56	1.5108	1.1076	0.0482	0.138*	
C57	1.3968 (17)	1.223 (4)	0.0655 (11)	0.125 (10)	
H57	1.4129	1.3175	0.0454	0.150*	
C58	1.3155 (18)	1.212 (4)	0.0946 (12)	0.107 (8)*	
H58	1.2767	1.3005	0.0972	0.129*	
C59	1.292 (2)	1.065 (3)	0.1202 (11)	0.104 (8)	
Cl1A	0.9494 (7)	0.4701 (16)	0.5565 (6)	0.110 (6)	0.509 (17)
O9A	0.905 (2)	0.692 (5)	0.4541 (17)	0.136 (13)*	0.509 (17)
C28A	0.856 (3)	0.696 (7)	0.495 (2)	0.117 (19)*	0.509 (17)
C29A	0.871 (3)	0.628 (6)	0.556 (2)	0.068 (17)*	0.509 (17)
H29A	0.8122	0.5882	0.5702	0.102*	0.509 (17)
H29B	0.8922	0.7105	0.5845	0.102*	0.509 (17)
O12A	0.956 (3)	1.500 (3)	0.3990 (17)	0.100 (17)	0.44 (4)
C36A	0.935 (5)	1.517 (8)	0.343 (3)	0.11 (3)*	0.44 (4)
C37A	0.926 (4)	1.667 (7)	0.317 (3)	0.06 (2)*	0.44 (4)
H37A	0.9310	1.7474	0.3497	0.092*	0.44 (4)
H37B	0.8674	1.6760	0.2960	0.092*	0.44 (4)
H37C	0.9742	1.6842	0.2881	0.092*	0.44 (4)
Cl1B	0.8673 (9)	0.645 (2)	0.5910 (8)	0.116 (6)	0.491 (17)
O9B	0.927 (2)	0.886 (5)	0.4500 (15)	0.127 (13)*	0.491 (17)
C28B	0.876 (3)	0.803 (8)	0.482 (3)	0.13 (2)*	0.491 (17)
C29B	0.932 (3)	0.705 (6)	0.529 (2)	0.110 (17)*	0.491 (17)
H29C	0.9839	0.7694	0.5443	0.164*	0.491 (17)
H29D	0.9567	0.6113	0.5080	0.164*	0.491 (17)
O12B	1.029 (2)	1.428 (4)	0.3464 (15)	0.130 (16)	0.56 (4)
C36B	0.956 (4)	1.486 (7)	0.334 (3)	0.11 (2)*	0.56 (4)
C37B	0.936 (5)	1.631 (8)	0.305 (3)	0.13 (3)*	0.56 (4)
H37D	0.9701	1.7152	0.3259	0.193*	0.56 (4)
H37E	0.8710	1.6526	0.3081	0.193*	0.56 (4)
H37F	0.9515	1.6260	0.2621	0.193*	0.56 (4)

### (OZTF) 4-Methoxyphenyl 4-O-[6-O-acetyl-2-azido-3-O-benzyl-2-deoxy-4-O-(9-fluorenylmethyloxycarbonyl)-α-D-glucopyranosyl]-2-O-benzoyl-3-O-benzyl-6-O-chloroacetyl-α-L-iodopyranoside Atomic displacement parameters (Å^2^)

**Table:**

	*U*^11^	*U*^22^	*U*^33^	*U*^12^	*U*^13^	*U*^23^
O1	0.120 (12)	0.077 (11)	0.065 (9)	0.009 (9)	0.023 (9)	0.018 (9)
O2	0.073 (10)	0.090 (11)	0.086 (11)	−0.008 (9)	0.001 (10)	0.007 (9)
O3	0.096 (11)	0.080 (11)	0.092 (10)	−0.003 (9)	0.038 (10)	0.011 (9)
O4	0.083 (10)	0.097 (14)	0.101 (11)	−0.024 (10)	0.007 (9)	−0.009 (10)
O5	0.090 (10)	0.100 (13)	0.088 (11)	0.014 (12)	0.026 (9)	−0.012 (11)
O6	0.104 (12)	0.126 (15)	0.088 (11)	−0.012 (12)	0.000 (10)	−0.007 (10)
O7	0.188 (17)	0.076 (12)	0.098 (11)	0.025 (12)	0.030 (11)	0.018 (10)
O8	0.111 (14)	0.112 (15)	0.131 (15)	0.025 (11)	−0.008 (11)	−0.020 (12)
O10	0.079 (12)	0.079 (12)	0.123 (13)	−0.026 (10)	0.013 (9)	−0.001 (11)
O11	0.081 (11)	0.108 (16)	0.140 (14)	0.002 (12)	−0.001 (9)	0.008 (13)
O13	0.186 (16)	0.097 (14)	0.106 (14)	−0.002 (12)	0.038 (12)	−0.006 (11)
O14	0.105 (13)	0.087 (12)	0.156 (14)	0.018 (11)	0.043 (10)	0.037 (12)
O15	0.106 (12)	0.097 (14)	0.134 (14)	0.013 (10)	0.003 (10)	0.014 (12)
O16	0.082 (11)	0.121 (14)	0.139 (13)	0.013 (11)	0.037 (10)	0.032 (12)
N1	0.23 (3)	0.14 (3)	0.084 (17)	−0.06 (2)	0.00 (2)	0.00 (2)
N2	0.17 (2)	0.14 (3)	0.16 (3)	−0.016 (18)	−0.01 (3)	−0.02 (3)
N3	0.103 (18)	0.19 (3)	0.22 (3)	−0.007 (18)	0.04 (2)	−0.04 (3)
C1	0.12 (2)	0.067 (18)	0.09 (2)	−0.005 (18)	0.02 (2)	−0.024 (17)
C2	0.086 (17)	0.08 (2)	0.10 (2)	0.012 (15)	−0.007 (17)	0.008 (16)
C3	0.071 (15)	0.11 (2)	0.062 (14)	−0.003 (17)	0.018 (13)	0.032 (15)
C4	0.087 (17)	0.063 (16)	0.097 (19)	−0.002 (14)	0.022 (17)	0.006 (16)
C5	0.098 (17)	0.12 (2)	0.054 (16)	−0.003 (19)	0.025 (14)	−0.005 (17)
C6	0.15 (2)	0.074 (17)	0.054 (15)	−0.030 (18)	0.033 (16)	−0.015 (15)
C7	0.148 (19)	0.063 (16)	0.055 (14)	0.013 (15)	0.017 (14)	0.013 (12)
C8	0.144 (19)	0.084 (17)	0.082 (16)	0.021 (18)	0.051 (15)	0.015 (15)
C9	0.13 (2)	0.11 (2)	0.11 (2)	0.05 (2)	0.013 (17)	−0.014 (19)
C10	0.12 (2)	0.14 (2)	0.096 (19)	0.032 (18)	0.018 (15)	−0.001 (17)
C11	0.103 (18)	0.100 (19)	0.084 (17)	−0.011 (17)	−0.004 (14)	−0.029 (15)
C13	0.08 (2)	0.13 (2)	0.10 (2)	−0.003 (18)	0.00 (2)	−0.019 (18)
C14	0.104 (19)	0.11 (2)	0.083 (19)	0.002 (16)	−0.003 (18)	−0.013 (16)
C15	0.11 (2)	0.13 (3)	0.10 (2)	0.013 (17)	−0.026 (17)	−0.013 (18)
C16	0.14 (2)	0.18 (3)	0.076 (17)	0.01 (2)	0.046 (17)	−0.018 (18)
C17	0.10 (2)	0.10 (2)	0.17 (3)	0.017 (18)	0.04 (2)	0.00 (2)
C18	0.12 (2)	0.086 (19)	0.087 (18)	0.003 (16)	0.007 (16)	0.001 (16)
C19	0.13 (2)	0.101 (19)	0.059 (15)	0.005 (17)	0.001 (15)	−0.018 (13)
C20	0.080 (17)	0.12 (3)	0.089 (18)	−0.015 (16)	0.001 (13)	0.047 (17)
C21	0.077 (16)	0.06 (2)	0.14 (3)	−0.006 (14)	0.012 (16)	0.03 (2)
C22	0.096 (19)	0.10 (3)	0.09 (2)	0.000 (19)	−0.003 (15)	−0.01 (2)
C23	0.061 (15)	0.14 (3)	0.12 (2)	−0.028 (17)	0.007 (13)	−0.01 (2)
C25	0.13 (2)	0.07 (2)	0.12 (2)	−0.019 (17)	0.014 (16)	−0.012 (18)
C26	0.11 (2)	0.13 (3)	0.12 (2)	−0.01 (2)	0.038 (17)	−0.02 (2)
C27	0.104 (19)	0.088 (19)	0.12 (2)	0.014 (18)	−0.021 (17)	−0.01 (2)
C30	0.12 (2)	0.07 (2)	0.10 (2)	0.02 (2)	0.028 (18)	0.042 (19)
C31	0.037 (14)	0.10 (2)	0.13 (2)	−0.003 (14)	0.006 (13)	0.018 (18)
C32	0.101 (19)	0.085 (19)	0.092 (18)	0.016 (18)	0.028 (16)	0.005 (16)
C33	0.15 (3)	0.07 (2)	0.09 (2)	−0.005 (19)	0.014 (19)	−0.006 (16)
C34	0.12 (2)	0.070 (18)	0.12 (2)	−0.031 (19)	0.012 (18)	−0.020 (18)
C35	0.098 (19)	0.11 (2)	0.104 (19)	−0.007 (18)	0.008 (15)	0.02 (2)
C39	0.10 (2)	0.13 (3)	0.10 (2)	0.01 (2)	0.032 (17)	−0.04 (2)
C40	0.13 (3)	0.14 (3)	0.14 (3)	0.01 (2)	0.05 (2)	0.02 (3)
C41	0.10 (2)	0.12 (3)	0.16 (4)	−0.02 (2)	0.03 (2)	−0.02 (2)
C42	0.12 (2)	0.12 (3)	0.15 (3)	0.00 (2)	0.00 (2)	0.00 (3)
C43	0.12 (2)	0.12 (3)	0.11 (3)	−0.01 (2)	0.015 (18)	−0.03 (2)
C44	0.13 (2)	0.10 (3)	0.14 (3)	0.04 (2)	−0.01 (2)	−0.02 (2)
C46	0.10 (2)	0.13 (3)	0.14 (2)	0.003 (19)	0.036 (18)	−0.03 (2)
C47	0.067 (17)	0.09 (2)	0.16 (3)	0.001 (16)	0.010 (17)	0.00 (2)
C48	0.10 (2)	0.12 (3)	0.097 (19)	0.00 (2)	0.015 (16)	−0.02 (2)
C49	0.16 (3)	0.10 (3)	0.13 (2)	−0.03 (3)	0.04 (2)	0.01 (2)
C50	0.15 (2)	0.10 (3)	0.077 (17)	0.01 (2)	−0.010 (15)	−0.013 (16)
C52	0.11 (2)	0.10 (2)	0.11 (2)	0.03 (2)	−0.031 (16)	−0.02 (2)
C53	0.09 (2)	0.12 (3)	0.077 (17)	−0.03 (2)	0.007 (15)	−0.005 (17)
C54	0.055 (17)	0.16 (3)	0.11 (2)	0.01 (2)	0.010 (15)	−0.04 (2)
C55	0.12 (2)	0.15 (3)	0.091 (18)	0.03 (2)	−0.018 (17)	−0.028 (19)
C57	0.104 (19)	0.16 (3)	0.11 (2)	0.00 (2)	0.023 (15)	−0.004 (19)
C59	0.10 (2)	0.10 (2)	0.110 (18)	0.02 (2)	−0.005 (16)	−0.010 (18)
Cl1A	0.088 (9)	0.101 (11)	0.140 (11)	0.007 (7)	−0.007 (7)	−0.031 (9)
O12A	0.15 (3)	0.05 (2)	0.11 (3)	−0.01 (2)	0.03 (2)	0.00 (2)
Cl1B	0.122 (11)	0.125 (13)	0.103 (13)	0.003 (9)	0.021 (9)	0.001 (11)
O12B	0.11 (3)	0.12 (3)	0.16 (3)	−0.02 (2)	0.01 (2)	0.02 (2)

### (OZTF) 4-Methoxyphenyl 4-O-[6-O-acetyl-2-azido-3-O-benzyl-2-deoxy-4-O-(9-fluorenylmethyloxycarbonyl)-α-D-glucopyranosyl]-2-O-benzoyl-3-O-benzyl-6-O-chloroacetyl-α-L-iodopyranoside Geometric parameters (Å, º)

**Table:**

O1—C1	1.38 (3)	C25—H25	0.9500
O1—C6	1.385 (19)	C26—H26	0.9500
O2—C13	1.33 (3)	C27—H27A	0.9900
O2—C2	1.47 (2)	C27—H27B	0.9900
O3—C3	1.44 (2)	C30—C34	1.56 (3)
O3—C20	1.47 (2)	C30—H30	1.0000
O4—C30	1.44 (3)	C31—C32	1.52 (3)
O4—C4	1.47 (2)	C31—C35	1.53 (3)
O5—C1	1.40 (3)	C31—H31	1.0000
O5—C5	1.44 (3)	C32—C33	1.54 (3)
O6—C13	1.25 (3)	C32—H32	1.0000
O7—C9	1.373 (19)	C33—C34	1.49 (3)
O7—C12	1.44 (2)	C33—H33	1.0000
O8—C28A	1.34 (4)	C34—H34	1.0000
O8—C28B	1.37 (4)	C35—H35A	0.9900
O8—C27	1.45 (3)	C35—H35B	0.9900
O10—C30	1.37 (3)	C38—C39	1.56 (4)
O10—C31	1.44 (2)	C38—H38A	0.9900
O11—C36B	1.30 (4)	C38—H38B	0.9900
O11—C36A	1.31 (4)	C39—C40	1.34 (4)
O11—C35	1.43 (3)	C39—C44	1.36 (4)
O13—C33	1.45 (3)	C40—C41	1.46 (4)
O13—C38	1.45 (4)	C40—H40	0.9500
O14—C45	1.39 (3)	C41—C42	1.35 (4)
O14—C32	1.44 (2)	C41—H41	0.9500
O15—C45	1.17 (3)	C42—C43	1.34 (4)
O16—C45	1.32 (4)	C42—H42	0.9500
O16—C46	1.44 (3)	C43—C44	1.40 (3)
N1—N2	1.18 (4)	C43—H43	0.9500
N1—C34	1.47 (3)	C44—H44	0.9500
N2—N3	1.19 (4)	C46—C47	1.46 (3)
C1—C2	1.53 (3)	C46—H46A	0.9900
C1—H1	1.0000	C46—H46B	0.9900
C2—C3	1.48 (3)	C47—C48	1.55 (4)
C2—H2	1.0000	C47—C59	1.59 (3)
C3—C4	1.52 (3)	C47—H47	1.0000
C3—H3	1.0000	C48—C49	1.40 (4)
C4—C5	1.51 (3)	C48—C53	1.40 (3)
C4—H4	1.0000	C49—C50	1.42 (4)
C5—C27	1.53 (3)	C49—H49	0.9500
C5—H5	1.0000	C50—C51	1.42 (3)
C6—C7	1.3900	C50—H50	0.9500
C6—C11	1.3900	C51—C52	1.37 (4)
C7—C8	1.3900	C51—H51	0.9500
C7—H7	0.9500	C52—C53	1.37 (3)
C8—C9	1.3900	C52—H52	0.9500
C8—H8	0.9500	C53—C54	1.52 (4)
C9—C10	1.3900	C54—C55	1.39 (3)
C10—C11	1.3900	C54—C59	1.39 (3)
C10—H10	0.9500	C55—C56	1.41 (4)
C11—H11	0.9500	C55—H55	0.9500
C12—H12A	0.9800	C56—C57	1.35 (4)
C12—H12B	0.9800	C56—H56	0.9500
C12—H12C	0.9800	C57—C58	1.38 (3)
C13—C14	1.50 (3)	C57—H57	0.9500
C14—C15	1.3900	C58—C59	1.41 (3)
C14—C19	1.3900	C58—H58	0.9500
C15—C16	1.3900	Cl1A—C29A	1.78 (4)
C15—H15	0.9500	O9A—C28A	1.17 (4)
C16—C17	1.3900	C28A—C29A	1.45 (4)
C16—H16	0.9500	C29A—H29A	0.9900
C17—C18	1.3900	C29A—H29B	0.9900
C17—H17	0.9500	O12A—C36A	1.25 (4)
C18—C19	1.3900	C36A—C37A	1.40 (5)
C18—H18	0.9500	C37A—H37A	0.9800
C19—H19	0.9500	C37A—H37B	0.9800
C20—C21	1.50 (3)	C37A—H37C	0.9800
C20—H20A	0.9900	Cl1B—C29B	1.77 (4)
C20—H20B	0.9900	O9B—C28B	1.27 (4)
C21—C26	1.37 (3)	C28B—C29B	1.53 (4)
C21—C22	1.37 (3)	C29B—H29C	0.9900
C22—C23	1.42 (4)	C29B—H29D	0.9900
C22—H22	0.9500	O12B—C36B	1.22 (4)
C23—C24	1.39 (3)	C36B—C37B	1.40 (5)
C23—H23	0.9500	C37B—H37D	0.9800
C24—C25	1.40 (3)	C37B—H37E	0.9800
C24—H24	0.9500	C37B—H37F	0.9800
C25—C26	1.35 (4)		
			
C1—O1—C6	119.9 (17)	O14—C32—C33	108 (2)
C13—O2—C2	112.6 (18)	C31—C32—C33	109 (2)
C3—O3—C20	115.3 (13)	O14—C32—H32	111.9
C30—O4—C4	117.8 (17)	C31—C32—H32	111.9
C1—O5—C5	111.1 (17)	C33—C32—H32	111.9
C9—O7—C12	116.5 (17)	O13—C33—C34	107 (2)
C28A—O8—C27	134 (3)	O13—C33—C32	106 (2)
C28B—O8—C27	105 (3)	C34—C33—C32	110 (2)
C30—O10—C31	115.7 (19)	O13—C33—H33	111.1
C36B—O11—C35	116 (3)	C34—C33—H33	111.1
C36A—O11—C35	125 (3)	C32—C33—H33	111.1
C33—O13—C38	115 (2)	N1—C34—C33	111 (3)
C45—O14—C32	117 (2)	N1—C34—C30	108 (2)
C45—O16—C46	114 (2)	C33—C34—C30	110 (2)
N2—N1—C34	116 (3)	N1—C34—H34	109.2
N1—N2—N3	170 (4)	C33—C34—H34	109.2
O1—C1—O5	114 (2)	C30—C34—H34	109.2
O1—C1—C2	110 (2)	O11—C35—C31	110 (2)
O5—C1—C2	113 (2)	O11—C35—H35A	109.7
O1—C1—H1	106.6	C31—C35—H35A	109.7
O5—C1—H1	106.6	O11—C35—H35B	109.7
C2—C1—H1	106.6	C31—C35—H35B	109.7
O2—C2—C3	110 (2)	H35A—C35—H35B	108.2
O2—C2—C1	102 (2)	O13—C38—C39	108 (3)
C3—C2—C1	113 (2)	O13—C38—H38A	110.1
O2—C2—H2	110.6	C39—C38—H38A	110.1
C3—C2—H2	110.6	O13—C38—H38B	110.1
C1—C2—H2	110.6	C39—C38—H38B	110.1
O3—C3—C2	108 (2)	H38A—C38—H38B	108.4
O3—C3—C4	107.1 (19)	C40—C39—C44	123 (3)
C2—C3—C4	115.0 (19)	C40—C39—C38	113 (4)
O3—C3—H3	109.0	C44—C39—C38	123 (4)
C2—C3—H3	109.0	C39—C40—C41	117 (3)
C4—C3—H3	109.0	C39—C40—H40	121.5
O4—C4—C5	111.4 (18)	C41—C40—H40	121.5
O4—C4—C3	108.3 (18)	C42—C41—C40	119 (3)
C5—C4—C3	112.5 (19)	C42—C41—H41	120.7
O4—C4—H4	108.2	C40—C41—H41	120.7
C5—C4—H4	108.2	C43—C42—C41	123 (4)
C3—C4—H4	108.2	C43—C42—H42	118.6
O5—C5—C4	106.5 (17)	C41—C42—H42	118.6
O5—C5—C27	110 (2)	C42—C43—C44	119 (3)
C4—C5—C27	111 (2)	C42—C43—H43	120.4
O5—C5—H5	109.8	C44—C43—H43	120.4
C4—C5—H5	109.8	C39—C44—C43	119 (3)
C27—C5—H5	109.8	C39—C44—H44	120.4
O1—C6—C7	116.6 (12)	C43—C44—H44	120.4
O1—C6—C11	123.4 (12)	O15—C45—O16	132 (3)
C7—C6—C11	120.0	O15—C45—O14	122 (3)
C8—C7—C6	120.0	O16—C45—O14	106 (3)
C8—C7—H7	120.0	O16—C46—C47	108 (2)
C6—C7—H7	120.0	O16—C46—H46A	110.1
C7—C8—C9	120.0	C47—C46—H46A	110.1
C7—C8—H8	120.0	O16—C46—H46B	110.1
C9—C8—H8	120.0	C47—C46—H46B	110.1
O7—C9—C10	127.0 (11)	H46A—C46—H46B	108.4
O7—C9—C8	113.0 (11)	C46—C47—C48	115 (2)
C10—C9—C8	120.0	C46—C47—C59	114 (2)
C11—C10—C9	120.0	C48—C47—C59	100 (2)
C11—C10—H10	120.0	C46—C47—H47	109.2
C9—C10—H10	120.0	C48—C47—H47	109.2
C10—C11—C6	120.0	C59—C47—H47	109.2
C10—C11—H11	120.0	C49—C48—C53	118 (3)
C6—C11—H11	120.0	C49—C48—C47	127 (3)
O7—C12—H12A	109.5	C53—C48—C47	115 (3)
O7—C12—H12B	109.5	C48—C49—C50	117 (3)
H12A—C12—H12B	109.5	C48—C49—H49	121.5
O7—C12—H12C	109.5	C50—C49—H49	121.5
H12A—C12—H12C	109.5	C51—C50—C49	121 (3)
H12B—C12—H12C	109.5	C51—C50—H50	119.7
O6—C13—O2	125 (2)	C49—C50—H50	119.7
O6—C13—C14	120 (3)	C52—C51—C50	123 (3)
O2—C13—C14	114 (2)	C52—C51—H51	118.7
C15—C14—C19	120.0	C50—C51—H51	118.7
C15—C14—C13	122.3 (15)	C53—C52—C51	115 (3)
C19—C14—C13	117.7 (15)	C53—C52—H52	122.5
C16—C15—C14	120.0	C51—C52—H52	122.5
C16—C15—H15	120.0	C52—C53—C48	126 (3)
C14—C15—H15	120.0	C52—C53—C54	130 (3)
C15—C16—C17	120.0	C48—C53—C54	104 (3)
C15—C16—H16	120.0	C55—C54—C59	115 (3)
C17—C16—H16	120.0	C55—C54—C53	131 (3)
C18—C17—C16	120.0	C59—C54—C53	113 (2)
C18—C17—H17	120.0	C54—C55—C56	122 (3)
C16—C17—H17	120.0	C54—C55—H55	119.0
C19—C18—C17	120.0	C56—C55—H55	119.0
C19—C18—H18	120.0	C57—C56—C55	121 (3)
C17—C18—H18	120.0	C57—C56—H56	119.6
C18—C19—C14	120.0	C55—C56—H56	119.6
C18—C19—H19	120.0	C56—C57—C58	120 (3)
C14—C19—H19	120.0	C56—C57—H57	120.2
O3—C20—C21	113.2 (19)	C58—C57—H57	120.2
O3—C20—H20A	108.9	C57—C58—C59	118 (3)
C21—C20—H20A	108.9	C57—C58—H58	120.8
O3—C20—H20B	108.9	C59—C58—H58	120.8
C21—C20—H20B	108.9	C54—C59—C58	124 (3)
H20A—C20—H20B	107.7	C54—C59—C47	108 (2)
C26—C21—C22	120 (3)	C58—C59—C47	128 (3)
C26—C21—C20	123 (3)	O9A—C28A—O8	115 (4)
C22—C21—C20	116 (3)	O9A—C28A—C29A	128 (4)
C21—C22—C23	120 (3)	O8—C28A—C29A	117 (4)
C21—C22—H22	119.8	C28A—C29A—Cl1A	113 (3)
C23—C22—H22	119.8	C28A—C29A—H29A	109.0
C24—C23—C22	118 (3)	Cl1A—C29A—H29A	109.0
C24—C23—H23	121.1	C28A—C29A—H29B	109.0
C22—C23—H23	121.1	Cl1A—C29A—H29B	109.0
C23—C24—C25	120 (3)	H29A—C29A—H29B	107.8
C23—C24—H24	120.0	O12A—C36A—O11	106 (4)
C25—C24—H24	120.0	O12A—C36A—C37A	121 (6)
C26—C25—C24	121 (3)	O11—C36A—C37A	125 (5)
C26—C25—H25	119.7	C36A—C37A—H37A	109.5
C24—C25—H25	119.7	C36A—C37A—H37B	109.5
C25—C26—C21	121 (3)	H37A—C37A—H37B	109.5
C25—C26—H26	119.7	C36A—C37A—H37C	109.5
C21—C26—H26	119.7	H37A—C37A—H37C	109.5
O8—C27—C5	106 (2)	H37B—C37A—H37C	109.5
O8—C27—H27A	110.5	O9B—C28B—O8	134 (5)
C5—C27—H27A	110.5	O9B—C28B—C29B	110 (4)
O8—C27—H27B	110.5	O8—C28B—C29B	115 (4)
C5—C27—H27B	110.5	C28B—C29B—Cl1B	111 (3)
H27A—C27—H27B	108.7	C28B—C29B—H29C	109.4
O10—C30—O4	112 (2)	Cl1B—C29B—H29C	109.4
O10—C30—C34	111 (2)	C28B—C29B—H29D	109.4
O4—C30—C34	105 (2)	Cl1B—C29B—H29D	109.4
O10—C30—H30	109.9	H29C—C29B—H29D	108.0
O4—C30—H30	109.9	O12B—C36B—O11	119 (5)
C34—C30—H30	109.9	O12B—C36B—C37B	129 (5)
O10—C31—C32	108.7 (19)	O11—C36B—C37B	109 (5)
O10—C31—C35	105 (2)	C36B—C37B—H37D	109.5
C32—C31—C35	115 (2)	C36B—C37B—H37E	109.5
O10—C31—H31	109.3	H37D—C37B—H37E	109.5
C32—C31—H31	109.3	C36B—C37B—H37F	109.5
C35—C31—H31	109.3	H37D—C37B—H37F	109.5
O14—C32—C31	103.3 (18)	H37E—C37B—H37F	109.5
			
C6—O1—C1—O5	−70 (2)	C31—C32—C33—C34	57 (3)
C6—O1—C1—C2	162.6 (18)	N2—N1—C34—C33	−82 (4)
C5—O5—C1—O1	−63 (2)	N2—N1—C34—C30	157 (3)
C5—O5—C1—C2	63 (2)	O13—C33—C34—N1	71 (3)
C13—O2—C2—C3	−85 (2)	C32—C33—C34—N1	−174 (2)
C13—O2—C2—C1	156 (2)	O13—C33—C34—C30	−169 (2)
O1—C1—C2—O2	−158.2 (18)	C32—C33—C34—C30	−54 (3)
O5—C1—C2—O2	73 (2)	O10—C30—C34—N1	175 (2)
O1—C1—C2—C3	84 (2)	O4—C30—C34—N1	55 (3)
O5—C1—C2—C3	−45 (3)	O10—C30—C34—C33	54 (3)
C20—O3—C3—C2	−121.3 (19)	O4—C30—C34—C33	−67 (3)
C20—O3—C3—C4	114.6 (18)	C36B—O11—C35—C31	−141 (4)
O2—C2—C3—O3	162.6 (16)	C36A—O11—C35—C31	−162 (5)
C1—C2—C3—O3	−84 (2)	O10—C31—C35—O11	−65 (2)
O2—C2—C3—C4	−78 (2)	C32—C31—C35—O11	54 (3)
C1—C2—C3—C4	35 (3)	C33—O13—C38—C39	169 (2)
C30—O4—C4—C5	−132 (2)	O13—C38—C39—C40	166 (3)
C30—O4—C4—C3	104 (2)	O13—C38—C39—C44	−15 (4)
O3—C3—C4—O4	−159.8 (17)	C44—C39—C40—C41	−2 (4)
C2—C3—C4—O4	81 (2)	C38—C39—C40—C41	177 (2)
O3—C3—C4—C5	77 (2)	C39—C40—C41—C42	2 (4)
C2—C3—C4—C5	−43 (3)	C40—C41—C42—C43	−1 (4)
C1—O5—C5—C4	−68 (2)	C41—C42—C43—C44	1 (4)
C1—O5—C5—C27	172 (2)	C40—C39—C44—C43	2 (4)
O4—C4—C5—O5	−65 (2)	C38—C39—C44—C43	−177 (3)
C3—C4—C5—O5	57 (2)	C42—C43—C44—C39	−2 (4)
O4—C4—C5—C27	55 (3)	C46—O16—C45—O15	−12 (4)
C3—C4—C5—C27	176.4 (19)	C46—O16—C45—O14	167 (2)
C1—O1—C6—C7	−155.0 (16)	C32—O14—C45—O15	−2 (4)
C1—O1—C6—C11	24 (2)	C32—O14—C45—O16	178.9 (19)
O1—C6—C7—C8	178.7 (12)	C45—O16—C46—C47	−98 (3)
C11—C6—C7—C8	0.0	O16—C46—C47—C48	−61 (3)
C6—C7—C8—C9	0.0	O16—C46—C47—C59	−176 (2)
C12—O7—C9—C10	1 (2)	C46—C47—C48—C49	59 (4)
C12—O7—C9—C8	179.8 (12)	C59—C47—C48—C49	−178 (2)
C7—C8—C9—O7	−179.2 (11)	C46—C47—C48—C53	−119 (2)
C7—C8—C9—C10	0.0	C59—C47—C48—C53	4 (3)
O7—C9—C10—C11	179.1 (13)	C53—C48—C49—C50	1 (4)
C8—C9—C10—C11	0.0	C47—C48—C49—C50	−177 (2)
C9—C10—C11—C6	0.0	C48—C49—C50—C51	0 (4)
O1—C6—C11—C10	−178.6 (12)	C49—C50—C51—C52	1 (4)
C7—C6—C11—C10	0.0	C50—C51—C52—C53	−3 (4)
C2—O2—C13—O6	3 (4)	C51—C52—C53—C48	4 (4)
C2—O2—C13—C14	−168.7 (19)	C51—C52—C53—C54	179 (2)
O6—C13—C14—C15	15 (3)	C49—C48—C53—C52	−3 (4)
O2—C13—C14—C15	−173.2 (15)	C47—C48—C53—C52	175 (2)
O6—C13—C14—C19	−167.5 (19)	C49—C48—C53—C54	−179 (2)
O2—C13—C14—C19	4 (3)	C47—C48—C53—C54	−1 (3)
C19—C14—C15—C16	0.0	C52—C53—C54—C55	5 (4)
C13—C14—C15—C16	177.6 (14)	C48—C53—C54—C55	−179 (2)
C14—C15—C16—C17	0.0	C52—C53—C54—C59	−179 (2)
C15—C16—C17—C18	0.0	C48—C53—C54—C59	−3 (3)
C16—C17—C18—C19	0.0	C59—C54—C55—C56	3 (3)
C17—C18—C19—C14	0.0	C53—C54—C55—C56	179 (2)
C15—C14—C19—C18	0.0	C54—C55—C56—C57	−4 (4)
C13—C14—C19—C18	−177.7 (13)	C55—C56—C57—C58	4 (4)
C3—O3—C20—C21	−81 (2)	C56—C57—C58—C59	−4 (4)
O3—C20—C21—C26	−84 (3)	C55—C54—C59—C58	−3 (4)
O3—C20—C21—C22	99 (2)	C53—C54—C59—C58	−180 (2)
C26—C21—C22—C23	6 (3)	C55—C54—C59—C47	−178 (2)
C20—C21—C22—C23	−177 (2)	C53—C54—C59—C47	5 (3)
C21—C22—C23—C24	−5 (3)	C57—C58—C59—C54	4 (4)
C22—C23—C24—C25	4 (4)	C57—C58—C59—C47	178 (2)
C23—C24—C25—C26	−3 (4)	C46—C47—C59—C54	118 (3)
C24—C25—C26—C21	4 (4)	C48—C47—C59—C54	−5 (3)
C22—C21—C26—C25	−5 (4)	C46—C47—C59—C58	−56 (3)
C20—C21—C26—C25	178 (2)	C48—C47—C59—C58	−180 (2)
C28A—O8—C27—C5	92 (4)	C28B—O8—C28A—O9A	−52 (5)
C28B—O8—C27—C5	129 (3)	C27—O8—C28A—O9A	7 (8)
O5—C5—C27—O8	−60 (2)	C28B—O8—C28A—C29A	122 (7)
C4—C5—C27—O8	−177.6 (19)	C27—O8—C28A—C29A	−179 (3)
C31—O10—C30—O4	58 (2)	O9A—C28A—C29A—Cl1A	−22 (8)
C31—O10—C30—C34	−58 (2)	O8—C28A—C29A—Cl1A	165 (4)
C4—O4—C30—O10	100 (2)	C36B—O11—C36A—O12A	−102 (14)
C4—O4—C30—C34	−141 (2)	C35—O11—C36A—O12A	−30 (10)
C30—O10—C31—C32	61 (2)	C36B—O11—C36A—C37A	110 (15)
C30—O10—C31—C35	−175.6 (18)	C35—O11—C36A—C37A	−179 (6)
C45—O14—C32—C31	−125 (2)	C28A—O8—C28B—O9B	151 (10)
C45—O14—C32—C33	119 (2)	C27—O8—C28B—O9B	11 (8)
O10—C31—C32—O14	−172.6 (18)	C28A—O8—C28B—C29B	−34 (4)
C35—C31—C32—O14	70 (3)	C27—O8—C28B—C29B	−174 (4)
O10—C31—C32—C33	−57 (3)	O9B—C28B—C29B—Cl1B	159 (4)
C35—C31—C32—C33	−175 (2)	O8—C28B—C29B—Cl1B	−17 (6)
C38—O13—C33—C34	−123 (2)	C36A—O11—C36B—O12B	139 (15)
C38—O13—C33—C32	120 (2)	C35—O11—C36B—O12B	19 (7)
O14—C32—C33—O13	−75 (2)	C36A—O11—C36B—C37B	−60 (11)
C31—C32—C33—O13	173 (2)	C35—O11—C36B—C37B	−179 (4)
O14—C32—C33—C34	169 (2)		

### (OZTF) 4-Methoxyphenyl 4-O-[6-O-acetyl-2-azido-3-O-benzyl-2-deoxy-4-O-(9-fluorenylmethyloxycarbonyl)-α-D-glucopyranosyl]-2-O-benzoyl-3-O-benzyl-6-O-chloroacetyl-α-L-iodopyranoside Hydrogen-bond geometry (Å, º)

Cg9 is the centroid of the C54–C59 ring.

**Table:**

*D*—H···*A*	*D*—H	H···*A*	*D*···*A*	*D*—H···*A*
C1—H1···O10^i^	1.00	2.53	3.51 (3)	169
C20—H20*A*···O7^ii^	0.99	2.57	3.44 (3)	146
C52—H52···O6^iii^	0.95	2.46	3.26 (3)	142
C16—H16···*Cg*9^iv^	0.95	2.65	3.520 (12)	152

Symmetry codes: (i) *x*, *y*−1, *z*; (ii) −*x*+1, *y*+1/2, −*z*+1; (iii) *x*+1, *y*, *z*; (iv) −*x*+2, *y*−1/2, −*z*.

### (RNSB) 4-Methoxyphenyl 4-O-[6-O-acetyl-2-azido-3-O-benzyl-2-deoxy-4-O-(9-fluorenylmethyloxycarbonyl)-α-D-glucopyranosyl]-2-O-benzoyl-3-O-benzyl-6-O-methoxyacetal-α-L-iodopyranoside Crystal data

**Table:**

C_60_H_59_N_3_O_17_	*F*(000) = 1152
*M**_r_* = 1094.10	*D*_x_ = 1.325 Mg m^−^^3^
Monoclinic, *P*2_1_	Cu *K*α radiation, λ = 1.54184 Å
*a* = 14.8595 (17) Å	Cell parameters from 3418 reflections
*b* = 8.3873 (6) Å	θ = 3.6–71.6°
*c* = 22.0138 (18) Å	µ = 0.81 mm^−^^1^
β = 90.939 (10)°	*T* = 120 K
*V* = 2743.2 (4) Å^3^	Plate, colourless
*Z* = 2	0.36 × 0.06 × 0.01 mm

### (RNSB) 4-Methoxyphenyl 4-O-[6-O-acetyl-2-azido-3-O-benzyl-2-deoxy-4-O-(9-fluorenylmethyloxycarbonyl)-α-D-glucopyranosyl]-2-O-benzoyl-3-O-benzyl-6-O-methoxyacetal-α-L-iodopyranoside Data collection

**Table:**

Agilent SuperNova (Dual, Cu at zero, Atlas) diffractometer	7922 independent reflections
Radiation source: SuperNova (Cu) X-ray Source	4977 reflections with *I* > 2σ(*I*)
Mirror monochromator	*R*_int_ = 0.101
Detector resolution: 5.3250 pixels mm^-1^	θ_max_ = 72.1°, θ_min_ = 5.0°
ω scans	*h* = −17→18
Absorption correction: gaussian (*CrysAlis PRO*; Agilent, 2013)	*k* = −10→7
*T*_min_ = 1.080, *T*_max_ = 1.638	*l* = −26→27
17226 measured reflections	

### (RNSB) 4-Methoxyphenyl 4-O-[6-O-acetyl-2-azido-3-O-benzyl-2-deoxy-4-O-(9-fluorenylmethyloxycarbonyl)-α-D-glucopyranosyl]-2-O-benzoyl-3-O-benzyl-6-O-methoxyacetal-α-L-iodopyranoside Refinement

**Table:**

Refinement on *F*^2^	Hydrogen site location: inferred from neighbouring sites
Least-squares matrix: full	H-atom parameters constrained
*R*[*F*^2^ > 2σ(*F*^2^)] = 0.083	*w* = 1/[σ^2^(*F*_o_^2^) + (0.0552*P*)^2^ + 2.9735*P*] where *P* = (*F*_o_^2^ + 2*F*_c_^2^)/3
*wR*(*F*^2^) = 0.201	(Δ/σ)_max_ < 0.001
*S* = 1.04	Δρ_max_ = 0.36 e Å^−^^3^
7922 reflections	Δρ_min_ = −0.32 e Å^−^^3^
730 parameters	Absolute structure: Flack *x* determined using 810 quotients [(*I*^+^)-(*I*^-^)]/[(*I*^+^)+(*I*^-^)] (Parsons & Flack, 2004)
38 restraints	Absolute structure parameter: −0.3 (4)

### (RNSB) 4-Methoxyphenyl 4-O-[6-O-acetyl-2-azido-3-O-benzyl-2-deoxy-4-O-(9-fluorenylmethyloxycarbonyl)-α-D-glucopyranosyl]-2-O-benzoyl-3-O-benzyl-6-O-methoxyacetal-α-L-iodopyranoside Special details

**Table:**

Geometry. All e.s.d.'s (except the e.s.d. in the dihedral angle between two l.s. planes) are estimated using the full covariance matrix. The cell e.s.d.'s are taken into account individually in the estimation of e.s.d.'s in distances, angles and torsion angles; correlations between e.s.d.'s in cell parameters are only used when they are defined by crystal symmetry. An approximate (isotropic) treatment of cell e.s.d.'s is used for estimating e.s.d.'s involving l.s. planes.

### (RNSB) 4-Methoxyphenyl 4-O-[6-O-acetyl-2-azido-3-O-benzyl-2-deoxy-4-O-(9-fluorenylmethyloxycarbonyl)-α-D-glucopyranosyl]-2-O-benzoyl-3-O-benzyl-6-O-methoxyacetal-α-L-iodopyranoside Fractional atomic coordinates and isotropic or equivalent isotropic displacement parameters (Å^2^)

**Table:**

	*x*	*y*	*z*	*U*_iso_*/*U*_eq_	Occ. (<1)
O1	0.5974 (4)	0.4383 (7)	0.4055 (2)	0.0312 (13)	
O2	0.6619 (3)	0.5309 (7)	0.2556 (2)	0.0285 (12)	
O3	0.4809 (4)	0.6979 (7)	0.3455 (3)	0.0330 (13)	
O4	0.6934 (4)	0.8710 (6)	0.2992 (2)	0.0301 (12)	
O5	0.7159 (3)	0.6128 (6)	0.3805 (2)	0.0304 (12)	
O6	0.5425 (4)	0.5104 (8)	0.1916 (3)	0.0414 (15)	
O7	0.7153 (5)	0.0322 (8)	0.5888 (3)	0.0494 (17)	
O8	0.7790 (5)	0.7824 (8)	0.4780 (3)	0.0507 (18)	
O9	0.9162 (6)	0.8544 (12)	0.4440 (4)	0.083 (3)	
O10	0.7396 (3)	1.1377 (6)	0.3065 (2)	0.0267 (12)	
O11	0.8736 (4)	1.3719 (6)	0.3201 (2)	0.0334 (13)	
O13	0.8389 (4)	0.9956 (7)	0.1422 (2)	0.0355 (14)	
O14	0.9591 (4)	1.0331 (7)	0.2470 (3)	0.0362 (13)	
O15	1.0172 (5)	1.2535 (8)	0.2043 (4)	0.0557 (19)	
O16	1.1008 (4)	1.0421 (8)	0.2336 (3)	0.0482 (16)	
O17	0.8426 (5)	0.6051 (11)	0.5627 (3)	0.067 (2)	
N1	0.6555 (5)	0.8991 (9)	0.1775 (3)	0.0397 (18)	
N2	0.6574 (5)	0.9015 (9)	0.1209 (4)	0.0394 (18)	
N3	0.6527 (6)	0.8888 (12)	0.0696 (4)	0.057 (2)	
C1	0.6589 (5)	0.4880 (9)	0.3605 (3)	0.0263 (17)	
H1	0.6965	0.3950	0.3481	0.032*	
C2	0.6005 (5)	0.5429 (9)	0.3057 (3)	0.0263 (16)	
H2	0.5502	0.4651	0.2993	0.032*	
C3	0.5615 (5)	0.7095 (9)	0.3105 (3)	0.0259 (16)	
H3	0.5458	0.7493	0.2689	0.031*	
C4	0.6247 (6)	0.8286 (10)	0.3418 (4)	0.0326 (19)	
H4	0.5903	0.9259	0.3537	0.039*	
C5	0.6682 (6)	0.7522 (9)	0.3984 (4)	0.0318 (19)	
H5	0.6210	0.7240	0.4284	0.038*	
C6	0.6339 (6)	0.3430 (10)	0.4517 (3)	0.0309 (18)	
C7	0.5736 (7)	0.2364 (10)	0.4784 (4)	0.040 (2)	
H7	0.5127	0.2325	0.4645	0.048*	
C8	0.6026 (7)	0.1366 (10)	0.5249 (4)	0.042 (2)	
H8	0.5610	0.0683	0.5445	0.050*	
C9	0.6919 (7)	0.1368 (11)	0.5428 (4)	0.039 (2)	
C10	0.7526 (6)	0.2435 (11)	0.5171 (4)	0.040 (2)	
H10	0.8134	0.2465	0.5310	0.048*	
C11	0.7230 (6)	0.3464 (10)	0.4705 (4)	0.0346 (19)	
H11	0.7640	0.4178	0.4521	0.042*	
C12	0.8074 (7)	0.0275 (13)	0.6083 (5)	0.060 (3)	
H12A	0.8455	0.0044	0.5735	0.089*	
H12B	0.8155	−0.0559	0.6391	0.089*	
H12C	0.8244	0.1309	0.6258	0.089*	
C13	0.6234 (5)	0.5117 (10)	0.1999 (3)	0.0289 (17)	
C14	0.6899 (6)	0.4813 (9)	0.1531 (3)	0.0309 (18)	
C15	0.6620 (6)	0.4724 (12)	0.0927 (4)	0.044 (2)	
H15	0.6002	0.4866	0.0823	0.053*	
C16	0.7236 (7)	0.4430 (13)	0.0473 (4)	0.051 (3)	
H16	0.7041	0.4374	0.0061	0.061*	
C17	0.8124 (7)	0.4220 (11)	0.0622 (4)	0.044 (2)	
H17	0.8540	0.4009	0.0309	0.052*	
C18	0.8434 (6)	0.4310 (11)	0.1225 (4)	0.038 (2)	
H18	0.9054	0.4176	0.1325	0.045*	
C19	0.7810 (6)	0.4598 (9)	0.1668 (4)	0.0328 (19)	
H19	0.8007	0.4651	0.2081	0.039*	
C20	0.4024 (5)	0.7609 (10)	0.3148 (4)	0.0328 (19)	
H20A	0.3478	0.7190	0.3345	0.039*	
H20B	0.4015	0.7237	0.2721	0.039*	
C21	0.3994 (5)	0.9398 (10)	0.3156 (4)	0.0300 (18)	
C22	0.4266 (6)	1.0325 (12)	0.2663 (4)	0.037 (2)	
H22	0.4464	0.9811	0.2304	0.044*	
C23	0.4254 (6)	1.1976 (11)	0.2686 (4)	0.040 (2)	
H23	0.4460	1.2581	0.2351	0.048*	
C24	0.3938 (6)	1.2745 (11)	0.3200 (4)	0.042 (2)	
H24	0.3919	1.3876	0.3214	0.050*	
C25	0.3651 (6)	1.1864 (11)	0.3689 (4)	0.043 (2)	
H25	0.3427	1.2391	0.4038	0.051*	
C26	0.3690 (6)	1.0197 (11)	0.3674 (4)	0.038 (2)	
H26	0.3508	0.9600	0.4018	0.045*	
C27	0.7366 (7)	0.8639 (12)	0.4270 (4)	0.053 (3)	
H27A	0.7824	0.8939	0.3969	0.063*	
H27B	0.7066	0.9623	0.4411	0.063*	
C28	0.8689 (8)	0.7805 (14)	0.4782 (5)	0.056 (3)	
C29	0.9102 (8)	0.6810 (15)	0.5294 (5)	0.065 (3)	
H29A	0.9508	0.5998	0.5121	0.078*	
H29B	0.9463	0.7506	0.5567	0.078*	
C30	0.6850 (5)	1.0246 (10)	0.2734 (3)	0.0313 (18)	
H30	0.6206	1.0588	0.2746	0.038*	
C31	0.8335 (5)	1.0987 (10)	0.3074 (4)	0.0309 (18)	
H31	0.8408	0.9902	0.3256	0.037*	
C32	0.8682 (5)	1.0938 (10)	0.2428 (4)	0.0317 (18)	
H32	0.8676	1.2029	0.2245	0.038*	
C33	0.8135 (5)	0.9788 (10)	0.2037 (3)	0.0315 (18)	
H33	0.8244	0.8669	0.2176	0.038*	
C34	0.7149 (5)	1.0187 (11)	0.2082 (3)	0.0341 (18)	
H34	0.7040	1.1254	0.1892	0.041*	
C35	0.8806 (6)	1.2169 (10)	0.3482 (4)	0.0326 (19)	
H35A	0.8521	1.2185	0.3885	0.039*	
H35B	0.9446	1.1869	0.3538	0.039*	
O12A	0.9654 (9)	1.4710 (10)	0.3913 (6)	0.073 (4)	0.797 (16)
C36A	0.9237 (8)	1.4888 (16)	0.3456 (6)	0.039 (3)	0.797 (16)
O12B	1.014 (3)	1.437 (5)	0.352 (2)	0.073 (4)	0.203 (16)
C36B	0.947 (3)	1.472 (6)	0.327 (2)	0.039 (3)	0.203 (16)
C37	0.9181 (7)	1.6388 (12)	0.3077 (5)	0.051 (3)	
H37A	0.9462	1.6207	0.2684	0.076*	
H37B	0.9493	1.7260	0.3290	0.076*	
H37C	0.8547	1.6672	0.3014	0.076*	
C38	0.8795 (8)	0.8584 (12)	0.1162 (4)	0.051 (3)	
H38A	0.8413	0.7638	0.1232	0.061*	
H38B	0.9390	0.8392	0.1358	0.061*	
C39	0.8911 (6)	0.8822 (10)	0.0489 (4)	0.036 (2)	
C40	0.9457 (6)	0.7760 (10)	0.0181 (4)	0.039 (2)	
H40	0.9769	0.6944	0.0397	0.047*	
C41	0.9546 (6)	0.7894 (10)	−0.0441 (4)	0.040 (2)	
H41	0.9902	0.7141	−0.0653	0.048*	
C42	0.9122 (6)	0.9115 (11)	−0.0759 (4)	0.040 (2)	
H42	0.9203	0.9227	−0.1184	0.048*	
C43	0.8576 (6)	1.0175 (12)	−0.0448 (4)	0.037 (2)	
H43	0.8264	1.0992	−0.0664	0.045*	
C44	0.8485 (6)	1.0046 (10)	0.0173 (4)	0.0348 (19)	
H44	0.8129	1.0798	0.0385	0.042*	
C45	1.0248 (6)	1.1229 (12)	0.2244 (4)	0.040 (2)	
C46	1.1809 (6)	1.0998 (11)	0.2032 (4)	0.043 (2)	
H46A	1.2330	1.0981	0.2317	0.051*	
H46B	1.1714	1.2110	0.1894	0.051*	
C47	1.1992 (6)	0.9954 (11)	0.1499 (4)	0.043 (2)	
H47	1.1477	1.0004	0.1202	0.051*	
C48	1.2186 (7)	0.8200 (10)	0.1676 (4)	0.039 (2)	
C49	1.1643 (8)	0.7166 (11)	0.2000 (5)	0.054 (3)	
H49	1.1065	0.7489	0.2131	0.064*	
C50	1.1972 (8)	0.5633 (12)	0.2128 (4)	0.052 (3)	
H50	1.1617	0.4908	0.2353	0.062*	
C51	1.2818 (7)	0.5166 (12)	0.1926 (4)	0.050 (3)	
H51	1.3029	0.4119	0.2011	0.060*	
C52	1.3359 (7)	0.6214 (12)	0.1599 (4)	0.048 (2)	
H52	1.3932	0.5887	0.1459	0.057*	
C53	1.3043 (6)	0.7746 (11)	0.1485 (4)	0.039 (2)	
C54	1.3456 (6)	0.9106 (10)	0.1179 (4)	0.0339 (19)	
C55	1.4293 (6)	0.9241 (13)	0.0914 (4)	0.048 (2)	
H55	1.4700	0.8368	0.0907	0.057*	
C56	1.4514 (7)	1.0717 (13)	0.0660 (5)	0.054 (3)	
H56	1.5085	1.0837	0.0477	0.064*	
C57	1.3945 (7)	1.1979 (14)	0.0664 (4)	0.049 (2)	
H57	1.4121	1.2963	0.0489	0.059*	
C58	1.3105 (7)	1.1834 (11)	0.0923 (4)	0.045 (2)	
H58	1.2700	1.2710	0.0922	0.053*	
C59	1.2862 (6)	1.0391 (12)	0.1186 (4)	0.039 (2)	
C60	0.8785 (8)	0.533 (2)	0.6169 (5)	0.083 (4)	
H60A	0.9276	0.4610	0.6065	0.124*	
H60B	0.8310	0.4734	0.6372	0.124*	
H60C	0.9015	0.6168	0.6442	0.124*	

### (RNSB) 4-Methoxyphenyl 4-O-[6-O-acetyl-2-azido-3-O-benzyl-2-deoxy-4-O-(9-fluorenylmethyloxycarbonyl)-α-D-glucopyranosyl]-2-O-benzoyl-3-O-benzyl-6-O-methoxyacetal-α-L-iodopyranoside Atomic displacement parameters (Å^2^)

**Table:**

	*U*^11^	*U*^22^	*U*^33^	*U*^12^	*U*^13^	*U*^23^
O1	0.039 (3)	0.025 (3)	0.029 (3)	−0.001 (3)	0.007 (2)	0.006 (2)
O2	0.038 (3)	0.023 (3)	0.025 (2)	0.001 (3)	0.004 (2)	−0.004 (2)
O3	0.030 (3)	0.030 (3)	0.039 (3)	0.008 (3)	0.008 (2)	0.003 (2)
O4	0.036 (3)	0.019 (3)	0.035 (3)	−0.003 (2)	0.007 (2)	0.004 (2)
O5	0.032 (3)	0.023 (3)	0.036 (3)	−0.002 (3)	0.000 (2)	0.001 (2)
O6	0.037 (3)	0.050 (4)	0.037 (3)	0.003 (3)	−0.001 (2)	−0.004 (3)
O7	0.077 (5)	0.031 (4)	0.041 (3)	0.015 (4)	0.007 (3)	0.012 (3)
O8	0.066 (5)	0.052 (4)	0.033 (3)	−0.013 (4)	−0.006 (3)	−0.001 (3)
O9	0.089 (6)	0.082 (7)	0.080 (6)	−0.008 (6)	0.016 (5)	0.015 (5)
O10	0.029 (3)	0.014 (3)	0.037 (3)	−0.002 (2)	0.001 (2)	0.001 (2)
O11	0.043 (3)	0.016 (3)	0.041 (3)	−0.003 (3)	0.001 (3)	−0.001 (2)
O13	0.053 (4)	0.021 (3)	0.033 (3)	0.002 (3)	0.003 (3)	−0.002 (2)
O14	0.039 (3)	0.023 (3)	0.046 (3)	0.003 (3)	0.010 (3)	0.009 (3)
O15	0.058 (5)	0.025 (4)	0.085 (5)	0.000 (3)	0.011 (4)	0.018 (4)
O16	0.042 (4)	0.037 (4)	0.066 (4)	0.008 (3)	0.014 (3)	0.006 (3)
O17	0.071 (5)	0.085 (6)	0.044 (4)	−0.023 (5)	−0.009 (4)	0.010 (4)
N1	0.046 (5)	0.034 (4)	0.039 (4)	−0.015 (4)	−0.002 (3)	−0.001 (3)
N2	0.034 (4)	0.034 (4)	0.051 (5)	−0.010 (3)	−0.003 (3)	−0.001 (3)
N3	0.053 (5)	0.080 (7)	0.037 (4)	−0.011 (5)	−0.003 (4)	−0.003 (4)
C1	0.034 (4)	0.019 (4)	0.026 (4)	−0.005 (3)	0.008 (3)	−0.003 (3)
C2	0.029 (4)	0.022 (4)	0.028 (4)	0.000 (3)	0.004 (3)	−0.001 (3)
C3	0.027 (4)	0.019 (4)	0.032 (4)	−0.004 (3)	0.007 (3)	−0.002 (3)
C4	0.039 (5)	0.024 (4)	0.035 (4)	−0.008 (4)	0.013 (4)	−0.004 (3)
C5	0.051 (5)	0.009 (4)	0.035 (4)	−0.003 (4)	0.002 (4)	0.000 (3)
C6	0.046 (5)	0.019 (4)	0.028 (4)	0.006 (4)	0.005 (3)	0.001 (3)
C7	0.054 (6)	0.026 (5)	0.040 (5)	−0.002 (4)	0.009 (4)	0.010 (4)
C8	0.062 (6)	0.020 (5)	0.044 (5)	−0.001 (4)	0.013 (5)	0.007 (4)
C9	0.062 (6)	0.031 (5)	0.026 (4)	0.007 (5)	0.004 (4)	0.011 (4)
C10	0.051 (6)	0.039 (5)	0.030 (4)	0.009 (4)	−0.004 (4)	0.000 (4)
C11	0.044 (5)	0.027 (5)	0.033 (4)	−0.001 (4)	0.007 (4)	0.003 (3)
C12	0.093 (8)	0.031 (5)	0.055 (6)	0.022 (6)	−0.008 (6)	0.009 (5)
C13	0.030 (2)	0.028 (2)	0.0291 (19)	0.0000 (12)	0.0006 (12)	0.0004 (12)
C14	0.046 (5)	0.018 (4)	0.029 (4)	0.005 (4)	0.001 (4)	0.002 (3)
C15	0.039 (5)	0.057 (7)	0.037 (5)	0.019 (5)	−0.003 (4)	−0.005 (4)
C16	0.068 (7)	0.059 (7)	0.026 (4)	0.017 (6)	0.004 (4)	−0.001 (4)
C17	0.063 (6)	0.027 (5)	0.041 (5)	−0.002 (5)	0.015 (5)	0.001 (4)
C18	0.044 (5)	0.033 (5)	0.036 (5)	0.004 (4)	0.010 (4)	0.006 (4)
C19	0.041 (5)	0.019 (4)	0.039 (4)	0.004 (4)	0.003 (4)	0.004 (3)
C20	0.024 (4)	0.029 (5)	0.046 (5)	−0.002 (4)	0.004 (4)	−0.002 (4)
C21	0.030 (4)	0.022 (4)	0.038 (4)	0.005 (3)	−0.001 (3)	−0.003 (3)
C22	0.037 (5)	0.045 (5)	0.028 (4)	0.004 (4)	−0.002 (3)	0.005 (4)
C23	0.039 (5)	0.033 (5)	0.048 (5)	0.006 (4)	−0.003 (4)	0.012 (4)
C24	0.053 (6)	0.019 (4)	0.054 (6)	0.003 (4)	−0.001 (5)	0.004 (4)
C25	0.046 (6)	0.035 (5)	0.047 (5)	0.012 (5)	0.006 (4)	−0.007 (4)
C26	0.048 (5)	0.030 (5)	0.035 (4)	0.004 (4)	0.006 (4)	−0.001 (4)
C27	0.086 (8)	0.040 (6)	0.031 (5)	−0.019 (6)	−0.009 (5)	−0.001 (4)
C28	0.075 (8)	0.054 (7)	0.040 (5)	−0.002 (6)	0.003 (5)	0.002 (5)
C29	0.070 (8)	0.063 (8)	0.062 (7)	−0.010 (6)	0.000 (6)	−0.008 (6)
C30	0.032 (2)	0.030 (2)	0.032 (2)	−0.0005 (12)	0.0005 (12)	0.0000 (12)
C31	0.037 (5)	0.019 (4)	0.036 (4)	0.000 (4)	0.000 (4)	0.001 (3)
C32	0.038 (5)	0.025 (4)	0.032 (4)	0.004 (4)	0.003 (3)	0.000 (3)
C33	0.033 (2)	0.030 (2)	0.031 (2)	0.0003 (12)	0.0010 (12)	−0.0001 (12)
C34	0.035 (2)	0.033 (2)	0.034 (2)	−0.0005 (12)	0.0005 (12)	0.0005 (12)
C35	0.037 (5)	0.024 (5)	0.037 (4)	0.002 (4)	−0.003 (4)	−0.006 (3)
O12A	0.111 (10)	0.026 (5)	0.081 (8)	−0.016 (5)	−0.054 (7)	0.003 (5)
C36A	0.039 (3)	0.038 (3)	0.039 (3)	−0.0001 (9)	0.0006 (9)	0.0000 (9)
O12B	0.111 (10)	0.026 (5)	0.081 (8)	−0.016 (5)	−0.054 (7)	0.003 (5)
C36B	0.039 (3)	0.038 (3)	0.039 (3)	−0.0001 (9)	0.0006 (9)	0.0000 (9)
C37	0.059 (6)	0.027 (5)	0.065 (6)	−0.013 (5)	−0.008 (5)	0.001 (5)
C38	0.084 (8)	0.031 (5)	0.039 (5)	0.027 (5)	0.010 (5)	0.002 (4)
C39	0.041 (5)	0.022 (4)	0.044 (5)	0.007 (4)	0.001 (4)	0.000 (4)
C40	0.042 (5)	0.022 (5)	0.053 (5)	0.015 (4)	0.007 (4)	0.005 (4)
C41	0.040 (5)	0.021 (5)	0.059 (6)	0.000 (4)	0.009 (4)	−0.006 (4)
C42	0.046 (5)	0.036 (5)	0.037 (4)	−0.003 (4)	0.000 (4)	−0.003 (4)
C43	0.042 (5)	0.034 (5)	0.037 (4)	0.001 (4)	−0.001 (4)	0.000 (4)
C44	0.045 (5)	0.019 (4)	0.040 (4)	−0.001 (4)	0.003 (4)	−0.001 (4)
C45	0.044 (5)	0.036 (6)	0.042 (5)	0.000 (5)	0.008 (4)	−0.010 (4)
C46	0.043 (5)	0.036 (5)	0.050 (5)	−0.003 (4)	0.019 (4)	−0.006 (4)
C47	0.050 (6)	0.032 (5)	0.047 (5)	0.005 (4)	0.008 (4)	−0.001 (4)
C48	0.058 (6)	0.022 (5)	0.037 (5)	−0.003 (4)	−0.002 (4)	−0.003 (4)
C49	0.079 (8)	0.025 (5)	0.056 (6)	−0.003 (5)	0.007 (5)	−0.004 (4)
C50	0.077 (8)	0.033 (6)	0.045 (5)	−0.004 (5)	−0.006 (5)	0.002 (4)
C51	0.067 (7)	0.020 (5)	0.062 (6)	0.008 (5)	−0.028 (5)	−0.005 (5)
C52	0.055 (6)	0.034 (6)	0.054 (6)	0.012 (5)	−0.016 (5)	−0.012 (5)
C53	0.045 (5)	0.039 (5)	0.032 (4)	0.007 (4)	−0.010 (4)	−0.008 (4)
C54	0.040 (5)	0.028 (5)	0.034 (4)	−0.001 (4)	−0.010 (4)	−0.002 (3)
C55	0.032 (5)	0.052 (7)	0.058 (6)	0.006 (5)	−0.004 (4)	−0.004 (5)
C56	0.045 (6)	0.058 (7)	0.057 (6)	−0.002 (5)	0.005 (5)	−0.001 (5)
C57	0.046 (6)	0.053 (6)	0.047 (6)	−0.006 (5)	0.004 (4)	0.007 (5)
C58	0.058 (6)	0.032 (5)	0.044 (5)	0.008 (5)	0.005 (5)	0.003 (4)
C59	0.035 (5)	0.047 (6)	0.036 (4)	−0.001 (4)	0.005 (4)	−0.006 (4)
C60	0.072 (8)	0.119 (12)	0.058 (7)	−0.004 (9)	−0.018 (6)	0.008 (8)

### (RNSB) 4-Methoxyphenyl 4-O-[6-O-acetyl-2-azido-3-O-benzyl-2-deoxy-4-O-(9-fluorenylmethyloxycarbonyl)-α-D-glucopyranosyl]-2-O-benzoyl-3-O-benzyl-6-O-methoxyacetal-α-L-iodopyranoside Geometric parameters (Å, º)

**Table:**

O1—C6	1.396 (9)	C23—C24	1.391 (13)
O1—C1	1.422 (9)	C23—H23	0.9500
O2—C13	1.353 (8)	C24—C25	1.379 (13)
O2—C2	1.447 (9)	C24—H24	0.9500
O3—C3	1.439 (9)	C25—C26	1.400 (13)
O3—C20	1.439 (9)	C25—H25	0.9500
O4—C30	1.412 (10)	C26—H26	0.9500
O4—C4	1.442 (9)	C27—H27A	0.9900
O5—C1	1.412 (9)	C27—H27B	0.9900
O5—C5	1.426 (10)	C28—C29	1.522 (15)
O6—C13	1.213 (9)	C29—H29A	0.9900
O7—C9	1.381 (9)	C29—H29B	0.9900
O7—C12	1.429 (12)	C30—C34	1.511 (10)
O8—C28	1.337 (13)	C30—H30	1.0000
O8—C27	1.449 (11)	C31—C35	1.503 (11)
O9—C28	1.209 (13)	C31—C32	1.521 (10)
O10—C31	1.432 (9)	C31—H31	1.0000
O10—C30	1.439 (9)	C32—C33	1.519 (11)
O11—C36A	1.348 (14)	C32—H32	1.0000
O11—C36B	1.38 (5)	C33—C34	1.508 (11)
O11—C35	1.442 (10)	C33—H33	1.0000
O13—C33	1.419 (9)	C34—H34	1.0000
O13—C38	1.424 (10)	C35—H35A	0.9900
O14—C45	1.336 (11)	C35—H35B	0.9900
O14—C32	1.445 (9)	O12A—C36A	1.182 (12)
O15—C45	1.186 (11)	C36A—C37	1.511 (15)
O16—C45	1.329 (11)	O12B—C36B	1.17 (3)
O16—C46	1.458 (10)	C36B—C37	1.52 (5)
O17—C29	1.406 (13)	C37—H37A	0.980
O17—C60	1.432 (13)	C37—H37B	0.981
N1—N2	1.246 (10)	C37—H37C	0.980
N1—C34	1.491 (11)	C38—C39	1.506 (12)
N2—N3	1.136 (10)	C38—H38A	0.9900
C1—C2	1.544 (10)	C38—H38B	0.9900
C1—H1	1.0000	C39—C44	1.387 (11)
C2—C3	1.517 (11)	C39—C40	1.390 (12)
C2—H2	1.0000	C40—C41	1.383 (13)
C3—C4	1.528 (11)	C40—H40	0.9500
C3—H3	1.0000	C41—C42	1.386 (12)
C4—C5	1.533 (11)	C41—H41	0.9500
C4—H4	1.0000	C42—C43	1.391 (12)
C5—C27	1.513 (12)	C42—H42	0.9500
C5—H5	1.0000	C43—C44	1.380 (11)
C6—C11	1.381 (11)	C43—H43	0.9500
C6—C7	1.402 (12)	C44—H44	0.9500
C7—C8	1.386 (12)	C46—C47	1.492 (12)
C7—H7	0.9500	C46—H46A	0.9900
C8—C9	1.377 (13)	C46—H46B	0.9900
C8—H8	0.9500	C47—C59	1.520 (12)
C9—C10	1.397 (13)	C47—C48	1.548 (12)
C10—C11	1.405 (11)	C47—H47	1.0000
C10—H10	0.9500	C48—C49	1.389 (13)
C11—H11	0.9500	C48—C53	1.400 (13)
C12—H12A	0.9800	C49—C50	1.402 (14)
C12—H12B	0.9800	C49—H49	0.9500
C12—H12C	0.9800	C50—C51	1.397 (14)
C13—C14	1.463 (11)	C50—H50	0.9500
C14—C15	1.388 (11)	C51—C52	1.399 (14)
C14—C19	1.393 (11)	C51—H51	0.9500
C15—C16	1.387 (13)	C52—C53	1.389 (13)
C15—H15	0.9500	C52—H52	0.9500
C16—C17	1.365 (13)	C53—C54	1.465 (12)
C16—H16	0.9500	C54—C55	1.385 (13)
C17—C18	1.400 (12)	C54—C59	1.393 (13)
C17—H17	0.9500	C55—C56	1.400 (15)
C18—C19	1.379 (12)	C55—H55	0.9500
C18—H18	0.9500	C56—C57	1.355 (14)
C19—H19	0.9500	C56—H56	0.9500
C20—C21	1.501 (11)	C57—C58	1.385 (13)
C20—H20A	0.9900	C57—H57	0.9500
C20—H20B	0.9900	C58—C59	1.392 (13)
C21—C22	1.400 (11)	C58—H58	0.9500
C21—C26	1.405 (11)	C60—H60A	0.9800
C22—C23	1.385 (13)	C60—H60B	0.9800
C22—H22	0.9500	C60—H60C	0.9800
			
C6—O1—C1	115.5 (6)	O4—C30—C34	109.1 (7)
C13—O2—C2	115.9 (6)	O10—C30—C34	109.3 (6)
C3—O3—C20	113.6 (6)	O4—C30—H30	109.3
C30—O4—C4	115.3 (6)	O10—C30—H30	109.3
C1—O5—C5	113.3 (6)	C34—C30—H30	109.3
C9—O7—C12	117.8 (8)	O10—C31—C35	107.5 (7)
C28—O8—C27	115.5 (8)	O10—C31—C32	109.8 (6)
C31—O10—C30	113.4 (6)	C35—C31—C32	114.5 (7)
C36A—O11—C36B	23 (2)	O10—C31—H31	108.3
C36A—O11—C35	116.2 (7)	C35—C31—H31	108.3
C36B—O11—C35	116.5 (18)	C32—C31—H31	108.3
C33—O13—C38	115.2 (6)	O14—C32—C33	107.7 (6)
C45—O14—C32	117.8 (7)	O14—C32—C31	106.3 (6)
C45—O16—C46	117.3 (8)	C33—C32—C31	111.1 (7)
C29—O17—C60	111.4 (9)	O14—C32—H32	110.5
N2—N1—C34	114.9 (7)	C33—C32—H32	110.5
N3—N2—N1	172.1 (9)	C31—C32—H32	110.5
O5—C1—O1	112.8 (6)	O13—C33—C34	108.4 (6)
O5—C1—C2	110.5 (6)	O13—C33—C32	109.2 (6)
O1—C1—C2	105.8 (6)	C34—C33—C32	109.5 (7)
O5—C1—H1	109.2	O13—C33—H33	109.9
O1—C1—H1	109.2	C34—C33—H33	109.9
C2—C1—H1	109.2	C32—C33—H33	109.9
O2—C2—C3	111.3 (6)	N1—C34—C33	112.9 (7)
O2—C2—C1	102.8 (6)	N1—C34—C30	105.8 (7)
C3—C2—C1	115.5 (6)	C33—C34—C30	111.7 (6)
O2—C2—H2	109.0	N1—C34—H34	108.7
C3—C2—H2	109.0	C33—C34—H34	108.7
C1—C2—H2	109.0	C30—C34—H34	108.7
O3—C3—C2	107.3 (6)	O11—C35—C31	108.0 (6)
O3—C3—C4	108.3 (6)	O11—C35—H35A	110.1
C2—C3—C4	113.7 (6)	C31—C35—H35A	110.1
O3—C3—H3	109.1	O11—C35—H35B	110.1
C2—C3—H3	109.1	C31—C35—H35B	110.1
C4—C3—H3	109.1	H35A—C35—H35B	108.4
O4—C4—C3	107.7 (6)	O12A—C36A—O11	122.7 (11)
O4—C4—C5	109.8 (7)	O12A—C36A—C37	126.8 (12)
C3—C4—C5	109.9 (6)	O11—C36A—C37	110.5 (8)
O4—C4—H4	109.8	O12B—C36B—O11	125 (4)
C3—C4—H4	109.8	O12B—C36B—C37	127 (4)
C5—C4—H4	109.8	O11—C36B—C37	108 (3)
O5—C5—C27	106.8 (7)	C36A—C37—C36B	21 (2)
O5—C5—C4	108.9 (6)	C36A—C37—H37A	109.8 (10)
C27—C5—C4	110.5 (7)	C36B—C37—H37A	89 (2)
O5—C5—H5	110.2	C36A—C37—H37B	109.7 (9)
C27—C5—H5	110.2	C36B—C37—H37B	114.9 (18)
C4—C5—H5	110.2	H37A—C37—H37B	109.4 (10)
C11—C6—O1	124.4 (7)	C36A—C37—H37C	109.0
C11—C6—C7	120.3 (8)	C36B—C37—H37C	122 (2)
O1—C6—C7	115.3 (7)	H37A—C37—H37C	109.5
C8—C7—C6	120.1 (9)	H37B—C37—H37C	109.4
C8—C7—H7	119.9	O13—C38—C39	110.1 (7)
C6—C7—H7	119.9	O13—C38—H38A	109.6
C9—C8—C7	119.7 (9)	C39—C38—H38A	109.6
C9—C8—H8	120.1	O13—C38—H38B	109.6
C7—C8—H8	120.1	C39—C38—H38B	109.6
C8—C9—O7	116.0 (8)	H38A—C38—H38B	108.1
C8—C9—C10	120.7 (8)	C44—C39—C40	119.6 (8)
O7—C9—C10	123.2 (9)	C44—C39—C38	122.2 (8)
C9—C10—C11	119.5 (8)	C40—C39—C38	118.2 (8)
C9—C10—H10	120.2	C41—C40—C39	119.9 (8)
C11—C10—H10	120.2	C41—C40—H40	120.1
C6—C11—C10	119.5 (8)	C39—C40—H40	120.1
C6—C11—H11	120.3	C40—C41—C42	120.6 (8)
C10—C11—H11	120.3	C40—C41—H41	119.7
O7—C12—H12A	109.5	C42—C41—H41	119.7
O7—C12—H12B	109.5	C41—C42—C43	119.3 (8)
H12A—C12—H12B	109.5	C41—C42—H42	120.4
O7—C12—H12C	109.5	C43—C42—H42	120.4
H12A—C12—H12C	109.5	C44—C43—C42	120.3 (8)
H12B—C12—H12C	109.5	C44—C43—H43	119.9
O6—C13—O2	122.7 (7)	C42—C43—H43	119.9
O6—C13—C14	124.9 (7)	C43—C44—C39	120.3 (8)
O2—C13—C14	112.2 (7)	C43—C44—H44	119.9
C15—C14—C19	118.3 (8)	C39—C44—H44	119.9
C15—C14—C13	119.3 (8)	O15—C45—O16	127.1 (9)
C19—C14—C13	122.3 (7)	O15—C45—O14	126.4 (9)
C16—C15—C14	120.6 (8)	O16—C45—O14	106.3 (8)
C16—C15—H15	119.7	O16—C46—C47	109.1 (8)
C14—C15—H15	119.7	O16—C46—H46A	109.9
C17—C16—C15	119.8 (8)	C47—C46—H46A	109.9
C17—C16—H16	120.1	O16—C46—H46B	109.9
C15—C16—H16	120.1	C47—C46—H46B	109.9
C16—C17—C18	121.5 (9)	H46A—C46—H46B	108.3
C16—C17—H17	119.3	C46—C47—C59	112.5 (8)
C18—C17—H17	119.3	C46—C47—C48	113.3 (8)
C19—C18—C17	117.7 (9)	C59—C47—C48	100.8 (8)
C19—C18—H18	121.1	C46—C47—H47	110.0
C17—C18—H18	121.1	C59—C47—H47	110.0
C18—C19—C14	122.1 (8)	C48—C47—H47	110.0
C18—C19—H19	119.0	C49—C48—C53	121.6 (9)
C14—C19—H19	119.0	C49—C48—C47	127.9 (9)
O3—C20—C21	112.7 (7)	C53—C48—C47	110.4 (8)
O3—C20—H20A	109.1	C48—C49—C50	118.1 (10)
C21—C20—H20A	109.1	C48—C49—H49	120.9
O3—C20—H20B	109.1	C50—C49—H49	120.9
C21—C20—H20B	109.1	C51—C50—C49	120.4 (10)
H20A—C20—H20B	107.8	C51—C50—H50	119.8
C22—C21—C26	117.8 (8)	C49—C50—H50	119.8
C22—C21—C20	122.5 (8)	C50—C51—C52	121.1 (9)
C26—C21—C20	119.7 (8)	C50—C51—H51	119.5
C23—C22—C21	121.5 (9)	C52—C51—H51	119.5
C23—C22—H22	119.3	C53—C52—C51	118.6 (9)
C21—C22—H22	119.3	C53—C52—H52	120.7
C22—C23—C24	119.9 (9)	C51—C52—H52	120.7
C22—C23—H23	120.1	C52—C53—C48	120.3 (9)
C24—C23—H23	120.1	C52—C53—C54	131.3 (9)
C25—C24—C23	120.0 (9)	C48—C53—C54	108.5 (8)
C25—C24—H24	120.0	C55—C54—C59	120.9 (9)
C23—C24—H24	120.0	C55—C54—C53	130.0 (9)
C24—C25—C26	120.2 (9)	C59—C54—C53	109.0 (8)
C24—C25—H25	119.9	C54—C55—C56	117.3 (9)
C26—C25—H25	119.9	C54—C55—H55	121.3
C25—C26—C21	120.6 (9)	C56—C55—H55	121.3
C25—C26—H26	119.7	C57—C56—C55	122.4 (10)
C21—C26—H26	119.7	C57—C56—H56	118.8
O8—C27—C5	108.1 (8)	C55—C56—H56	118.8
O8—C27—H27A	110.1	C56—C57—C58	120.1 (10)
C5—C27—H27A	110.1	C56—C57—H57	120.0
O8—C27—H27B	110.1	C58—C57—H57	120.0
C5—C27—H27B	110.1	C57—C58—C59	119.3 (9)
H27A—C27—H27B	108.4	C57—C58—H58	120.4
O9—C28—O8	125.6 (11)	C59—C58—H58	120.4
O9—C28—C29	120.7 (11)	C58—C59—C54	120.0 (8)
O8—C28—C29	113.6 (9)	C58—C59—C47	128.8 (9)
O17—C29—C28	110.6 (9)	C54—C59—C47	111.3 (8)
O17—C29—H29A	109.5	O17—C60—H60A	109.5
C28—C29—H29A	109.5	O17—C60—H60B	109.5
O17—C29—H29B	109.5	H60A—C60—H60B	109.5
C28—C29—H29B	109.5	O17—C60—H60C	109.5
H29A—C29—H29B	108.1	H60A—C60—H60C	109.5
O4—C30—O10	110.6 (6)	H60B—C60—H60C	109.5
			
C5—O5—C1—O1	−59.2 (8)	C35—C31—C32—C33	−176.6 (7)
C5—O5—C1—C2	59.0 (8)	C38—O13—C33—C34	−124.8 (8)
C6—O1—C1—O5	−76.6 (8)	C38—O13—C33—C32	116.0 (8)
C6—O1—C1—C2	162.5 (6)	O14—C32—C33—O13	−72.7 (8)
C13—O2—C2—C3	−80.3 (8)	C31—C32—C33—O13	171.2 (6)
C13—O2—C2—C1	155.5 (6)	O14—C32—C33—C34	168.8 (6)
O5—C1—C2—O2	79.3 (7)	C31—C32—C33—C34	52.7 (9)
O1—C1—C2—O2	−158.3 (6)	N2—N1—C34—C33	−71.6 (10)
O5—C1—C2—C3	−42.1 (9)	N2—N1—C34—C30	165.8 (7)
O1—C1—C2—C3	80.3 (7)	O13—C33—C34—N1	68.2 (9)
C20—O3—C3—C2	−124.4 (7)	C32—C33—C34—N1	−172.8 (6)
C20—O3—C3—C4	112.5 (7)	O13—C33—C34—C30	−172.6 (7)
O2—C2—C3—O3	160.2 (6)	C32—C33—C34—C30	−53.6 (9)
C1—C2—C3—O3	−83.1 (8)	O4—C30—C34—N1	58.7 (8)
O2—C2—C3—C4	−80.1 (8)	O10—C30—C34—N1	179.8 (6)
C1—C2—C3—C4	36.6 (9)	O4—C30—C34—C33	−64.6 (9)
C30—O4—C4—C3	105.9 (7)	O10—C30—C34—C33	56.5 (9)
C30—O4—C4—C5	−134.5 (6)	C36A—O11—C35—C31	−170.3 (9)
O3—C3—C4—O4	−165.1 (6)	C36B—O11—C35—C31	−144 (3)
C2—C3—C4—O4	75.7 (8)	O10—C31—C35—O11	−66.1 (8)
O3—C3—C4—C5	75.3 (8)	C32—C31—C35—O11	56.2 (9)
C2—C3—C4—C5	−43.8 (9)	C36B—O11—C36A—O12A	−103 (5)
C1—O5—C5—C27	171.8 (6)	C35—O11—C36A—O12A	−6.1 (17)
C1—O5—C5—C4	−68.8 (8)	C36B—O11—C36A—C37	77 (5)
O4—C4—C5—O5	−59.9 (8)	C35—O11—C36A—C37	173.6 (9)
C3—C4—C5—O5	58.4 (9)	C36A—O11—C36B—O12B	99 (8)
O4—C4—C5—C27	57.1 (9)	C35—O11—C36B—O12B	3 (6)
C3—C4—C5—C27	175.4 (7)	C36A—O11—C36B—C37	−72 (5)
C1—O1—C6—C11	27.0 (11)	C35—O11—C36B—C37	−167.0 (19)
C1—O1—C6—C7	−151.6 (7)	O12A—C36A—C37—C36B	102 (5)
C11—C6—C7—C8	1.7 (13)	O11—C36A—C37—C36B	−78 (5)
O1—C6—C7—C8	−179.7 (8)	O12B—C36B—C37—C36A	−101 (8)
C6—C7—C8—C9	−3.3 (14)	O11—C36B—C37—C36A	70 (5)
C7—C8—C9—O7	−179.1 (8)	C33—O13—C38—C39	171.8 (7)
C7—C8—C9—C10	4.0 (14)	O13—C38—C39—C44	−13.6 (13)
C12—O7—C9—C8	179.4 (8)	O13—C38—C39—C40	167.1 (8)
C12—O7—C9—C10	−3.8 (13)	C44—C39—C40—C41	−2.4 (14)
C8—C9—C10—C11	−3.1 (14)	C38—C39—C40—C41	176.9 (9)
O7—C9—C10—C11	−179.7 (8)	C39—C40—C41—C42	2.4 (14)
O1—C6—C11—C10	−179.3 (8)	C40—C41—C42—C43	−2.4 (13)
C7—C6—C11—C10	−0.8 (13)	C41—C42—C43—C44	2.4 (13)
C9—C10—C11—C6	1.4 (13)	C42—C43—C44—C39	−2.4 (13)
C2—O2—C13—O6	2.7 (12)	C40—C39—C44—C43	2.4 (13)
C2—O2—C13—C14	−173.0 (6)	C38—C39—C44—C43	−176.9 (9)
O6—C13—C14—C15	9.8 (14)	C46—O16—C45—O15	−16.1 (14)
O2—C13—C14—C15	−174.5 (8)	C46—O16—C45—O14	169.0 (7)
O6—C13—C14—C19	−169.8 (8)	C32—O14—C45—O15	5.2 (13)
O2—C13—C14—C19	5.9 (11)	C32—O14—C45—O16	−179.9 (6)
C19—C14—C15—C16	−0.1 (14)	C45—O16—C46—C47	−103.4 (9)
C13—C14—C15—C16	−179.7 (9)	O16—C46—C47—C59	−175.3 (7)
C14—C15—C16—C17	0.2 (16)	O16—C46—C47—C48	−61.8 (10)
C15—C16—C17—C18	−0.6 (16)	C46—C47—C48—C49	56.9 (13)
C16—C17—C18—C19	0.8 (14)	C59—C47—C48—C49	177.3 (9)
C17—C18—C19—C14	−0.7 (13)	C46—C47—C48—C53	−120.5 (9)
C15—C14—C19—C18	0.3 (13)	C59—C47—C48—C53	−0.1 (9)
C13—C14—C19—C18	179.9 (8)	C53—C48—C49—C50	−0.6 (14)
C3—O3—C20—C21	−76.9 (8)	C47—C48—C49—C50	−177.8 (9)
O3—C20—C21—C22	98.8 (9)	C48—C49—C50—C51	−0.8 (14)
O3—C20—C21—C26	−80.9 (10)	C49—C50—C51—C52	0.8 (14)
C26—C21—C22—C23	1.2 (12)	C50—C51—C52—C53	0.7 (13)
C20—C21—C22—C23	−178.4 (8)	C51—C52—C53—C48	−2.1 (12)
C21—C22—C23—C24	−2.1 (14)	C51—C52—C53—C54	177.8 (8)
C22—C23—C24—C25	1.0 (14)	C49—C48—C53—C52	2.1 (13)
C23—C24—C25—C26	0.9 (14)	C47—C48—C53—C52	179.7 (7)
C24—C25—C26—C21	−1.8 (15)	C49—C48—C53—C54	−177.9 (8)
C22—C21—C26—C25	0.8 (13)	C47—C48—C53—C54	−0.3 (10)
C20—C21—C26—C25	−179.6 (8)	C52—C53—C54—C55	−0.1 (15)
C28—O8—C27—C5	127.8 (9)	C48—C53—C54—C55	179.9 (9)
O5—C5—C27—O8	−58.5 (9)	C52—C53—C54—C59	−179.5 (9)
C4—C5—C27—O8	−176.8 (7)	C48—C53—C54—C59	0.5 (9)
C27—O8—C28—O9	8.6 (16)	C59—C54—C55—C56	0.3 (13)
C27—O8—C28—C29	−174.8 (9)	C53—C54—C55—C56	−179.1 (9)
C60—O17—C29—C28	−170.1 (10)	C54—C55—C56—C57	−0.1 (15)
O9—C28—C29—O17	−179.5 (11)	C55—C56—C57—C58	−0.5 (16)
O8—C28—C29—O17	3.7 (14)	C56—C57—C58—C59	1.0 (15)
C4—O4—C30—O10	95.6 (7)	C57—C58—C59—C54	−0.8 (14)
C4—O4—C30—C34	−144.2 (6)	C57—C58—C59—C47	179.4 (9)
C31—O10—C30—O4	59.7 (8)	C55—C54—C59—C58	0.2 (13)
C31—O10—C30—C34	−60.4 (8)	C53—C54—C59—C58	179.7 (8)
C30—O10—C31—C35	−174.6 (6)	C55—C54—C59—C47	180.0 (8)
C30—O10—C31—C32	60.2 (8)	C53—C54—C59—C47	−0.6 (10)
C45—O14—C32—C33	117.8 (8)	C46—C47—C59—C58	−58.9 (13)
C45—O14—C32—C31	−123.1 (7)	C48—C47—C59—C58	−179.9 (9)
O10—C31—C32—O14	−172.4 (6)	C46—C47—C59—C54	121.4 (9)
C35—C31—C32—O14	66.5 (9)	C48—C47—C59—C54	0.4 (9)
O10—C31—C32—C33	−55.5 (8)		

### (RNSB) 4-Methoxyphenyl 4-O-[6-O-acetyl-2-azido-3-O-benzyl-2-deoxy-4-O-(9-fluorenylmethyloxycarbonyl)-α-D-glucopyranosyl]-2-O-benzoyl-3-O-benzyl-6-O-methoxyacetal-α-L-iodopyranoside Hydrogen-bond geometry (Å, º)

**Table:**

*D*—H···*A*	*D*—H	H···*A*	*D*···*A*	*D*—H···*A*
C1—H1···O10^i^	1.00	2.43	3.395 (9)	161
C3—H3···O6	1.00	2.63	3.114 (10)	110
C11—H11···O5	0.95	2.37	2.987 (10)	122
C20—H20*A*···O7^ii^	0.99	2.50	3.370 (11)	147
C29—H29*B*···O12*A*^iii^	0.99	2.53	3.503 (14)	168
C29—H29*B*···O12*B*^iii^	0.99	2.60	3.55 (4)	159

Symmetry codes: (i) *x*, *y*−1, *z*; (ii) −*x*+1, *y*+1/2, −*z*+1; (iii) −*x*+2, *y*−1/2, −*z*+1.

### (RSTN) 4-Methoxyphenyl 4-O-[6-O-acetyl-2-azido-3,4-O-benzyl-2-deoxy-α-D-glucopyranosyl]-2-O-benzoyl-3-O-benzyl-6-O-methoxyoacetyl-β-D-glucopyranoside Crystal data

**Table:**

C_52_H_55_N_3_O_15_	*F*(000) = 2032
*M**_r_* = 961.99	*D*_x_ = 1.277 Mg m^−^^3^
Monoclinic, *C*2	Mo *K*α radiation, λ = 0.71073 Å
Hall symbol: C 2y	Cell parameters from 9943 reflections
*a* = 38.3346 (13) Å	θ = 2.5–26.0°
*b* = 8.0744 (3) Å	µ = 0.09 mm^−^^1^
*c* = 16.1659 (6) Å	*T* = 118 K
β = 91.222 (2)°	Block, colourless
*V* = 5002.7 (3) Å^3^	0.75 × 0.32 × 0.30 mm
*Z* = 4	

### (RSTN) 4-Methoxyphenyl 4-O-[6-O-acetyl-2-azido-3,4-O-benzyl-2-deoxy-α-D-glucopyranosyl]-2-O-benzoyl-3-O-benzyl-6-O-methoxyoacetyl-β-D-glucopyranoside Data collection

**Table:**

Bruker APEXII CCD diffractometer	9796 independent reflections
Radiation source: fine-focus sealed tube	9128 reflections with *I* > 2σ(*I*)
Graphite monochromator	*R*_int_ = 0.035
φ and ω scans	θ_max_ = 26.1°, θ_min_ = 2.6°
Absorption correction: multi-scan (Blessing, 1995)	*h* = −47→47
*T*_min_ = 0.645, *T*_max_ = 0.745	*k* = −9→9
51621 measured reflections	*l* = −19→19

### (RSTN) 4-Methoxyphenyl 4-O-[6-O-acetyl-2-azido-3,4-O-benzyl-2-deoxy-α-D-glucopyranosyl]-2-O-benzoyl-3-O-benzyl-6-O-methoxyoacetyl-β-D-glucopyranoside Refinement

**Table:**

Refinement on *F*^2^	Secondary atom site location: difference Fourier map
Least-squares matrix: full	Hydrogen site location: inferred from neighbouring sites
*R*[*F*^2^ > 2σ(*F*^2^)] = 0.048	H-atom parameters constrained
*wR*(*F*^2^) = 0.129	*w* = 1/[σ^2^(*F*_o_^2^) + (0.0693*P*)^2^ + 3.3214*P*] where *P* = (*F*_o_^2^ + 2*F*_c_^2^)/3
*S* = 1.08	(Δ/σ)_max_ < 0.001
9796 reflections	Δρ_max_ = 0.33 e Å^−^^3^
662 parameters	Δρ_min_ = −0.45 e Å^−^^3^
43 restraints	Absolute structure: Flack *x* determined using 3878 quotients [(*I*^+^)-(*I*^-^)]/[(*I*^+^)+(*I*^-^)] (Parsons & Flack, 2004)
Primary atom site location: structure-invariant direct methods	Absolute structure parameter: 0.0 (2)

### (RSTN) 4-Methoxyphenyl 4-O-[6-O-acetyl-2-azido-3,4-O-benzyl-2-deoxy-α-D-glucopyranosyl]-2-O-benzoyl-3-O-benzyl-6-O-methoxyoacetyl-β-D-glucopyranoside Special details

**Table:**

Geometry. All e.s.d.'s (except the e.s.d. in the dihedral angle between two l.s. planes) are estimated using the full covariance matrix. The cell e.s.d.'s are taken into account individually in the estimation of e.s.d.'s in distances, angles and torsion angles; correlations between e.s.d.'s in cell parameters are only used when they are defined by crystal symmetry. An approximate (isotropic) treatment of cell e.s.d.'s is used for estimating e.s.d.'s involving l.s. planes.

### (RSTN) 4-Methoxyphenyl 4-O-[6-O-acetyl-2-azido-3,4-O-benzyl-2-deoxy-α-D-glucopyranosyl]-2-O-benzoyl-3-O-benzyl-6-O-methoxyoacetyl-β-D-glucopyranoside Fractional atomic coordinates and isotropic or equivalent isotropic displacement parameters (Å^2^)

**Table:**

	*x*	*y*	*z*	*U*_iso_*/*U*_eq_	Occ. (<1)
O1	0.21921 (5)	0.4100 (3)	−0.00970 (14)	0.0277 (5)	
O2	0.22146 (6)	0.5216 (4)	0.15747 (16)	0.0412 (6)	
O3	0.28436 (6)	0.4516 (3)	0.23969 (13)	0.0278 (5)	
O4	0.34523 (5)	0.5269 (3)	0.14741 (13)	0.0236 (4)	
O5	0.27881 (5)	0.4256 (2)	−0.01262 (14)	0.0251 (5)	
O7	0.16256 (7)	0.5146 (3)	−0.32199 (15)	0.0394 (6)	
O8	0.35123 (6)	0.3047 (3)	−0.02746 (14)	0.0286 (5)	
O9	0.39653 (6)	0.3629 (3)	−0.10867 (16)	0.0409 (6)	
O10	0.40059 (5)	0.4825 (3)	0.09354 (13)	0.0260 (5)	
O11	0.41635 (8)	0.7054 (3)	−0.03756 (15)	0.0416 (6)	
O12	0.46316 (12)	0.6820 (5)	−0.1148 (2)	0.0751 (11)	
O13	0.41911 (6)	0.6246 (3)	0.33709 (14)	0.0314 (5)	
O14	0.43940 (5)	0.8397 (3)	0.20138 (14)	0.0272 (5)	
N1	0.36688 (8)	0.3804 (4)	0.29835 (18)	0.0337 (6)	
N2	0.38032 (8)	0.3150 (4)	0.36028 (19)	0.0356 (7)	
N3	0.38900 (9)	0.2514 (5)	0.4196 (2)	0.0492 (8)	
C1	0.24830 (7)	0.4906 (4)	0.02421 (19)	0.0248 (6)	
H1	0.2466	0.6130	0.0157	0.030*	
C2	0.25012 (8)	0.4482 (4)	0.1158 (2)	0.0280 (7)	
H2	0.2490	0.3251	0.1224	0.034*	
C3	0.28340 (7)	0.5116 (4)	0.15736 (19)	0.0241 (6)	
H3	0.2830	0.6354	0.1582	0.029*	
C4	0.31510 (7)	0.4529 (4)	0.10944 (18)	0.0223 (6)	
H4	0.3170	0.3295	0.1125	0.027*	
C5	0.30969 (7)	0.5071 (4)	0.01892 (19)	0.0239 (6)	
H5	0.3051	0.6290	0.0185	0.029*	
C6	0.20745 (8)	0.4557 (4)	−0.0883 (2)	0.0261 (6)	
C7	0.17153 (8)	0.4415 (4)	−0.1024 (2)	0.0292 (7)	
H7	0.1568	0.4137	−0.0580	0.035*	
C8	0.15727 (8)	0.4677 (4)	−0.1806 (2)	0.0308 (7)	
H8	0.1327	0.4599	−0.1895	0.037*	
C9	0.17874 (9)	0.5054 (4)	−0.2464 (2)	0.0307 (7)	
C10	0.21433 (9)	0.5270 (4)	−0.2315 (2)	0.0329 (7)	
H10	0.2289	0.5599	−0.2753	0.040*	
C11	0.22865 (8)	0.5007 (4)	−0.1525 (2)	0.0309 (7)	
H11	0.2530	0.5136	−0.1430	0.037*	
C12	0.18366 (12)	0.5427 (6)	−0.3918 (3)	0.0516 (10)	
H12A	0.2009	0.4535	−0.3961	0.077*	
H12B	0.1958	0.6490	−0.3855	0.077*	
H12C	0.1689	0.5450	−0.4421	0.077*	
C20	0.29003 (10)	0.5768 (4)	0.3010 (2)	0.0351 (8)	
H20A	0.2727	0.6666	0.2934	0.042*	
H20B	0.3136	0.6250	0.2954	0.042*	
C21	0.28663 (10)	0.5012 (5)	0.3852 (2)	0.0366 (8)	
C22	0.31276 (11)	0.5203 (6)	0.4449 (2)	0.0485 (10)	
H22	0.3330	0.5827	0.4325	0.058*	
C23	0.30977 (14)	0.4495 (7)	0.5228 (3)	0.0657 (13)	
H23	0.3277	0.4652	0.5635	0.079*	
C24	0.28092 (15)	0.3570 (7)	0.5409 (3)	0.0658 (14)	
H24	0.2790	0.3071	0.5939	0.079*	
C25	0.25468 (14)	0.3365 (6)	0.4823 (3)	0.0600 (12)	
H25	0.2347	0.2723	0.4949	0.072*	
C26	0.25716 (11)	0.4088 (5)	0.4050 (3)	0.0463 (9)	
H26	0.2387	0.3954	0.3653	0.056*	
C27	0.33921 (8)	0.4737 (4)	−0.03852 (19)	0.0263 (6)	
H27A	0.3312	0.4907	−0.0965	0.032*	
H27B	0.3586	0.5516	−0.0266	0.032*	
C28	0.38152 (9)	0.2704 (4)	−0.0636 (2)	0.0335 (7)	
C30	0.37582 (8)	0.4299 (4)	0.15163 (19)	0.0251 (6)	
H30	0.3695	0.3119	0.1400	0.030*	
C31	0.41278 (8)	0.6510 (4)	0.10516 (19)	0.0256 (6)	
H31	0.3925	0.7284	0.0998	0.031*	
C32	0.42956 (8)	0.6702 (4)	0.1908 (2)	0.0259 (6)	
H32	0.4508	0.5986	0.1952	0.031*	
C33	0.40369 (8)	0.6187 (4)	0.25641 (19)	0.0264 (6)	
H33	0.3829	0.6933	0.2537	0.032*	
C34	0.39242 (8)	0.4411 (4)	0.23799 (19)	0.0274 (6)	
H34	0.4135	0.3679	0.2405	0.033*	
C35	0.43757 (9)	0.6842 (5)	0.0365 (2)	0.0346 (8)	
H35A	0.4513	0.7855	0.0483	0.041*	
H35B	0.4538	0.5900	0.0302	0.041*	
C36	0.43232 (15)	0.7026 (5)	−0.1094 (3)	0.0547 (12)	
C37	0.40762 (19)	0.7282 (8)	−0.1807 (3)	0.0823 (19)	
H37A	0.4112	0.6418	−0.2222	0.123*	
H37B	0.4118	0.8371	−0.2054	0.123*	
H37C	0.3836	0.7227	−0.1614	0.123*	
C38	0.40767 (9)	0.7630 (5)	0.3857 (2)	0.0353 (8)	
H38A	0.3820	0.7592	0.3909	0.042*	
H38B	0.4139	0.8679	0.3579	0.042*	
C39	0.42460 (10)	0.7562 (6)	0.4689 (2)	0.0437 (9)	
C40	0.44197 (13)	0.8939 (7)	0.5015 (3)	0.0597 (12)	
H40	0.4433	0.9916	0.4690	0.072*	
C41	0.45723 (13)	0.8930 (8)	0.5793 (3)	0.0690 (14)	
H41	0.4694	0.9874	0.5999	0.083*	
C42	0.45440 (15)	0.7490 (8)	0.6274 (3)	0.0739 (15)	
H42	0.4643	0.7455	0.6818	0.089*	
C43	0.43734 (18)	0.6141 (10)	0.5956 (4)	0.096 (2)*	
H43	0.4360	0.5160	0.6279	0.115*	
C44	0.42186 (18)	0.6169 (7)	0.5172 (3)	0.0794 (18)	
H44	0.4094	0.5229	0.4970	0.095*	
C45	0.47356 (8)	0.8549 (4)	0.2404 (2)	0.0316 (7)	
H45A	0.4907	0.7904	0.2089	0.038*	
H45B	0.4730	0.8098	0.2973	0.038*	
C46	0.48429 (8)	1.0337 (4)	0.2432 (2)	0.0293 (7)	
C47	0.51154 (10)	1.0881 (5)	0.1951 (3)	0.0482 (10)	
H47	0.5226	1.0128	0.1590	0.058*	
C48	0.52278 (12)	1.2495 (6)	0.1991 (4)	0.0699 (15)	
H48	0.5414	1.2849	0.1656	0.084*	
C49	0.50726 (10)	1.3595 (5)	0.2512 (3)	0.0571 (12)	
H49	0.5149	1.4714	0.2529	0.069*	
C50	0.48048 (11)	1.3084 (5)	0.3014 (3)	0.0513 (11)	
H50	0.4701	1.3838	0.3387	0.062*	
C51	0.46894 (10)	1.1447 (5)	0.2964 (2)	0.0396 (8)	
H51	0.4503	1.1091	0.3300	0.047*	
C29A	0.3951 (2)	0.1011 (9)	−0.0349 (8)	0.041 (2)	0.687 (8)
H29A	0.3916	0.0908	0.0254	0.062*	0.687 (8)
H29B	0.3810	0.0136	−0.0627	0.062*	0.687 (8)
O52A	0.43001 (10)	0.0741 (5)	−0.0510 (3)	0.0469 (13)	0.687 (8)
C52A	0.4526 (2)	0.1588 (14)	0.0038 (6)	0.083 (3)	0.687 (8)
H52A	0.4414	0.1724	0.0572	0.125*	0.687 (8)
H52B	0.4580	0.2679	−0.0191	0.125*	0.687 (8)
H52C	0.4742	0.0951	0.0115	0.125*	0.687 (8)
O6A	0.20348 (12)	0.2611 (5)	0.1990 (3)	0.0627 (14)	0.793 (6)
C13A	0.20088 (14)	0.4076 (8)	0.2016 (3)	0.0375 (12)	0.793 (6)
C14A	0.17567 (5)	0.4975 (4)	0.25286 (14)	0.0447 (14)	0.793 (6)
C15A	0.15873 (7)	0.4088 (5)	0.3137 (2)	0.069 (2)	0.793 (6)
H15A	0.1639	0.2948	0.3220	0.083*	0.793 (6)
C16A	0.13420 (8)	0.4867 (6)	0.3625 (2)	0.088 (3)	0.793 (6)
H16A	0.1226	0.4260	0.4041	0.105*	0.793 (6)
C17A	0.12662 (6)	0.6534 (5)	0.35032 (18)	0.088 (3)	0.793 (6)
H17A	0.1099	0.7066	0.3836	0.105*	0.793 (6)
C18A	0.14357 (7)	0.7421 (5)	0.2894 (2)	0.085 (3)	0.793 (6)
H18A	0.1384	0.8561	0.2811	0.102*	0.793 (6)
C19A	0.16809 (7)	0.6642 (5)	0.2407 (2)	0.0523 (16)	0.793 (6)
H19A	0.1797	0.7249	0.1991	0.063*	0.793 (6)
C29B	0.39001 (13)	0.0897 (9)	−0.0557 (4)	0.051 (9)	0.313 (8)
H29C	0.3691	0.0211	−0.0672	0.077*	0.313 (8)
H29D	0.4085	0.0579	−0.0946	0.077*	0.313 (8)
O52B	0.40174 (11)	0.0699 (8)	0.0271 (4)	0.034 (2)	0.313 (8)
C52B	0.43802 (10)	0.1337 (9)	0.0431 (5)	0.040 (3)	0.313 (8)
H52D	0.4549	0.0583	0.0183	0.061*	0.313 (8)
H52E	0.4426	0.1405	0.1029	0.061*	0.313 (8)
H52F	0.4403	0.2440	0.0185	0.061*	0.313 (8)
O6B	0.18867 (7)	0.3442 (6)	0.1734 (2)	0.062 (6)	0.207 (6)
C13B	0.19440 (6)	0.4882 (5)	0.18013 (14)	0.048 (6)	0.207 (6)
C14B	0.1715 (4)	0.6106 (16)	0.2229 (10)	0.050 (7)*	0.207 (6)
C15B	0.1407 (5)	0.5567 (18)	0.2577 (13)	0.081 (8)*	0.207 (6)
H15B	0.1341	0.4435	0.2537	0.097*	0.207 (6)
C16B	0.1194 (4)	0.669 (2)	0.2984 (14)	0.108 (12)*	0.207 (6)
H16B	0.0984	0.6317	0.3222	0.129*	0.207 (6)
C17B	0.1291 (4)	0.834 (2)	0.3042 (12)	0.088 (9)*	0.207 (6)
H17B	0.1145	0.9106	0.3320	0.105*	0.207 (6)
C18B	0.1599 (4)	0.8880 (15)	0.2694 (10)	0.054 (5)*	0.207 (6)
H18B	0.1665	1.0012	0.2733	0.065*	0.207 (6)
C19B	0.1811 (3)	0.7762 (17)	0.2287 (8)	0.032 (4)*	0.207 (6)
H19B	0.2022	0.8130	0.2049	0.038*	0.207 (6)

### (RSTN) 4-Methoxyphenyl 4-O-[6-O-acetyl-2-azido-3,4-O-benzyl-2-deoxy-α-D-glucopyranosyl]-2-O-benzoyl-3-O-benzyl-6-O-methoxyoacetyl-β-D-glucopyranoside Atomic displacement parameters (Å^2^)

**Table:**

	*U*^11^	*U*^22^	*U*^33^	*U*^12^	*U*^13^	*U*^23^
O1	0.0233 (11)	0.0214 (11)	0.0385 (13)	−0.0042 (8)	−0.0001 (9)	0.0024 (9)
O2	0.0239 (12)	0.0550 (17)	0.0450 (15)	0.0024 (12)	0.0088 (10)	−0.0133 (12)
O3	0.0342 (11)	0.0212 (10)	0.0283 (11)	−0.0034 (9)	0.0066 (9)	0.0002 (9)
O4	0.0230 (10)	0.0198 (10)	0.0282 (11)	−0.0009 (8)	0.0022 (8)	−0.0015 (8)
O5	0.0223 (10)	0.0202 (11)	0.0330 (11)	0.0012 (8)	0.0019 (8)	−0.0036 (9)
O7	0.0448 (14)	0.0360 (13)	0.0373 (13)	−0.0002 (11)	−0.0020 (11)	−0.0005 (11)
O8	0.0273 (11)	0.0229 (11)	0.0359 (12)	0.0027 (9)	0.0063 (9)	−0.0031 (9)
O9	0.0383 (13)	0.0345 (14)	0.0508 (15)	0.0030 (11)	0.0183 (12)	−0.0014 (12)
O10	0.0260 (10)	0.0214 (11)	0.0309 (11)	0.0005 (8)	0.0063 (8)	−0.0031 (9)
O11	0.0598 (17)	0.0382 (14)	0.0272 (13)	−0.0113 (12)	0.0097 (11)	−0.0035 (10)
O12	0.107 (3)	0.060 (2)	0.061 (2)	−0.009 (2)	0.053 (2)	−0.0009 (16)
O13	0.0329 (12)	0.0332 (12)	0.0279 (12)	0.0037 (10)	−0.0017 (9)	−0.0001 (10)
O14	0.0230 (10)	0.0231 (11)	0.0356 (12)	−0.0001 (9)	−0.0006 (9)	−0.0019 (9)
N1	0.0359 (15)	0.0339 (15)	0.0315 (15)	−0.0015 (12)	0.0039 (12)	0.0076 (12)
N2	0.0373 (15)	0.0322 (15)	0.0374 (17)	−0.0057 (12)	0.0001 (13)	0.0048 (13)
N3	0.053 (2)	0.055 (2)	0.0397 (18)	−0.0120 (17)	−0.0055 (15)	0.0162 (17)
C1	0.0206 (13)	0.0163 (14)	0.0376 (17)	0.0010 (11)	0.0031 (12)	−0.0010 (12)
C2	0.0258 (15)	0.0219 (15)	0.0367 (17)	−0.0028 (12)	0.0105 (13)	−0.0027 (13)
C3	0.0242 (14)	0.0179 (14)	0.0304 (16)	−0.0005 (12)	0.0056 (12)	−0.0011 (12)
C4	0.0237 (14)	0.0152 (13)	0.0280 (15)	−0.0007 (11)	0.0032 (11)	−0.0009 (11)
C5	0.0226 (14)	0.0176 (13)	0.0317 (16)	−0.0003 (11)	0.0034 (12)	−0.0004 (12)
C6	0.0267 (15)	0.0133 (13)	0.0382 (17)	0.0005 (11)	0.0019 (12)	−0.0015 (12)
C7	0.0253 (15)	0.0219 (15)	0.0406 (18)	−0.0014 (12)	0.0046 (13)	−0.0007 (13)
C8	0.0249 (15)	0.0241 (16)	0.0433 (19)	−0.0026 (12)	0.0003 (13)	−0.0014 (14)
C9	0.0377 (17)	0.0161 (14)	0.0382 (18)	0.0030 (13)	−0.0017 (14)	−0.0007 (13)
C10	0.0336 (17)	0.0253 (16)	0.0403 (19)	0.0003 (14)	0.0089 (14)	0.0042 (14)
C11	0.0235 (15)	0.0225 (16)	0.047 (2)	0.0005 (12)	0.0026 (13)	0.0024 (14)
C12	0.058 (3)	0.056 (3)	0.040 (2)	0.006 (2)	0.0034 (18)	0.0027 (19)
C20	0.046 (2)	0.0258 (16)	0.0340 (18)	−0.0034 (14)	0.0089 (15)	−0.0054 (14)
C21	0.0444 (19)	0.0325 (18)	0.0334 (18)	0.0038 (15)	0.0136 (15)	−0.0051 (14)
C22	0.049 (2)	0.055 (3)	0.041 (2)	0.000 (2)	0.0118 (17)	−0.0040 (18)
C23	0.074 (3)	0.087 (4)	0.037 (2)	0.016 (3)	0.004 (2)	0.002 (2)
C24	0.090 (4)	0.071 (3)	0.037 (2)	0.011 (3)	0.024 (2)	0.012 (2)
C25	0.071 (3)	0.056 (3)	0.054 (3)	−0.004 (2)	0.037 (2)	0.004 (2)
C26	0.047 (2)	0.051 (2)	0.042 (2)	−0.0016 (18)	0.0168 (17)	−0.0029 (18)
C27	0.0276 (15)	0.0242 (15)	0.0273 (15)	0.0038 (12)	0.0044 (12)	−0.0008 (12)
C28	0.0289 (16)	0.0287 (17)	0.0433 (19)	0.0005 (13)	0.0120 (14)	−0.0068 (15)
C30	0.0235 (14)	0.0209 (15)	0.0309 (16)	0.0020 (12)	0.0052 (12)	0.0003 (12)
C31	0.0249 (14)	0.0213 (15)	0.0309 (16)	0.0004 (12)	0.0055 (12)	−0.0008 (12)
C32	0.0216 (14)	0.0243 (15)	0.0319 (17)	0.0021 (12)	0.0017 (12)	−0.0007 (12)
C33	0.0256 (15)	0.0258 (16)	0.0276 (16)	0.0015 (12)	−0.0017 (12)	0.0000 (13)
C34	0.0252 (15)	0.0258 (16)	0.0312 (16)	0.0008 (13)	0.0026 (12)	0.0021 (13)
C35	0.0391 (18)	0.0318 (17)	0.0331 (18)	−0.0053 (15)	0.0108 (15)	−0.0054 (14)
C36	0.092 (4)	0.034 (2)	0.039 (2)	−0.019 (2)	0.032 (2)	−0.0042 (17)
C37	0.150 (6)	0.071 (4)	0.026 (2)	−0.031 (4)	0.015 (3)	0.001 (2)
C38	0.0375 (18)	0.0384 (19)	0.0300 (17)	0.0028 (15)	0.0036 (14)	−0.0018 (14)
C39	0.039 (2)	0.056 (2)	0.037 (2)	0.0041 (18)	0.0022 (16)	−0.0038 (18)
C40	0.066 (3)	0.063 (3)	0.051 (3)	−0.017 (2)	−0.001 (2)	0.001 (2)
C41	0.064 (3)	0.091 (4)	0.051 (3)	−0.020 (3)	−0.011 (2)	−0.005 (3)
C42	0.073 (3)	0.092 (4)	0.056 (3)	−0.017 (3)	−0.023 (3)	0.008 (3)
C44	0.133 (5)	0.058 (3)	0.046 (3)	−0.017 (3)	−0.031 (3)	0.008 (2)
C45	0.0250 (16)	0.0292 (17)	0.0406 (18)	0.0001 (13)	0.0006 (13)	0.0021 (14)
C46	0.0222 (15)	0.0314 (17)	0.0342 (17)	−0.0009 (13)	−0.0018 (13)	−0.0008 (14)
C47	0.039 (2)	0.035 (2)	0.072 (3)	−0.0031 (16)	0.0223 (19)	−0.0058 (19)
C48	0.048 (3)	0.045 (2)	0.118 (5)	−0.017 (2)	0.032 (3)	−0.004 (3)
C49	0.037 (2)	0.030 (2)	0.104 (4)	−0.0043 (17)	−0.005 (2)	−0.013 (2)
C50	0.051 (2)	0.041 (2)	0.062 (3)	0.0125 (18)	−0.007 (2)	−0.0182 (19)
C51	0.0396 (19)	0.037 (2)	0.042 (2)	0.0033 (16)	0.0040 (16)	0.0006 (16)
C29A	0.035 (3)	0.024 (4)	0.064 (5)	0.004 (3)	0.021 (4)	0.004 (3)
O52A	0.038 (2)	0.037 (2)	0.067 (3)	0.0119 (17)	0.0163 (19)	−0.0014 (19)
C52A	0.061 (5)	0.093 (7)	0.095 (7)	0.033 (5)	−0.015 (5)	−0.020 (6)
O6A	0.051 (3)	0.043 (3)	0.095 (4)	−0.0131 (19)	0.035 (2)	0.010 (2)
C13A	0.025 (2)	0.053 (4)	0.035 (3)	−0.003 (2)	0.006 (2)	0.006 (2)
C14A	0.0194 (19)	0.080 (4)	0.035 (2)	−0.006 (2)	0.0021 (17)	−0.008 (2)
C15A	0.049 (3)	0.106 (6)	0.053 (3)	−0.026 (3)	0.020 (3)	−0.005 (3)
C16A	0.047 (3)	0.159 (8)	0.058 (4)	−0.027 (4)	0.028 (3)	−0.026 (5)
C17A	0.042 (3)	0.168 (9)	0.054 (4)	0.015 (4)	0.010 (3)	−0.038 (5)
C18A	0.076 (5)	0.127 (7)	0.052 (4)	0.047 (5)	0.003 (3)	−0.015 (4)
C19A	0.045 (3)	0.078 (5)	0.034 (3)	0.018 (3)	0.002 (2)	−0.011 (3)
C29B	0.035 (10)	0.050 (13)	0.069 (15)	0.003 (8)	0.007 (8)	−0.016 (9)
O52B	0.027 (4)	0.038 (5)	0.038 (5)	0.008 (3)	0.010 (3)	0.005 (4)
C52B	0.034 (7)	0.032 (6)	0.055 (9)	0.009 (5)	−0.006 (6)	−0.003 (6)
O6B	0.043 (10)	0.063 (13)	0.082 (13)	−0.037 (9)	0.025 (9)	−0.011 (10)
C13B	0.028 (10)	0.052 (15)	0.064 (15)	−0.022 (10)	−0.015 (9)	−0.001 (11)

### (RSTN) 4-Methoxyphenyl 4-O-[6-O-acetyl-2-azido-3,4-O-benzyl-2-deoxy-α-D-glucopyranosyl]-2-O-benzoyl-3-O-benzyl-6-O-methoxyoacetyl-β-D-glucopyranoside Geometric parameters (Å, º)

**Table:**

O1—C6	1.389 (4)	C33—H33	1.0000
O1—C1	1.393 (4)	C34—H34	1.0000
O2—C13B	1.140 (3)	C35—H35A	0.9900
O2—C13A	1.415 (6)	C35—H35B	0.9900
O2—C2	1.430 (4)	C36—C37	1.490 (8)
O3—C3	1.416 (4)	C37—H37A	0.9800
O3—C20	1.430 (4)	C37—H37B	0.9800
O4—C30	1.410 (4)	C37—H37C	0.9800
O4—C4	1.428 (4)	C38—C39	1.482 (5)
O5—C1	1.424 (3)	C38—H38A	0.9900
O5—C5	1.438 (4)	C38—H38B	0.9900
O7—C9	1.361 (4)	C39—C44	1.374 (7)
O7—C12	1.421 (5)	C39—C40	1.394 (6)
O8—C28	1.340 (4)	C40—C41	1.376 (7)
O8—C27	1.450 (4)	C40—H40	0.9500
O9—C28	1.199 (4)	C41—C42	1.404 (8)
O10—C30	1.415 (4)	C41—H41	0.9500
O10—C31	1.449 (4)	C42—C43	1.366 (9)
O11—C36	1.325 (5)	C42—H42	0.9500
O11—C35	1.443 (5)	C43—C44	1.389 (8)
O12—C36	1.199 (7)	C43—H43	0.9500
O13—C33	1.421 (4)	C44—H44	0.9500
O13—C38	1.440 (4)	C45—C46	1.501 (5)
O14—C32	1.429 (4)	C45—H45A	0.9900
O14—C45	1.446 (4)	C45—H45B	0.9900
N1—N2	1.235 (4)	C46—C51	1.382 (5)
N1—C34	1.480 (4)	C46—C47	1.387 (5)
N2—N3	1.132 (4)	C47—C48	1.373 (6)
C1—C2	1.520 (4)	C47—H47	0.9500
C1—H1	1.0000	C48—C49	1.369 (7)
C2—C3	1.518 (4)	C48—H48	0.9500
C2—H2	1.0000	C49—C50	1.385 (7)
C3—C4	1.530 (4)	C49—H49	0.9500
C3—H3	1.0000	C50—C51	1.396 (6)
C4—C5	1.537 (4)	C50—H50	0.9500
C4—H4	1.0000	C51—H51	0.9500
C5—C27	1.503 (4)	C29A—O52A	1.386 (8)
C5—H5	1.0000	C29A—H29A	0.9900
C6—C11	1.381 (5)	C29A—H29B	0.9900
C6—C7	1.396 (4)	O52A—C52A	1.403 (10)
C7—C8	1.384 (5)	C52A—H52A	0.9800
C7—H7	0.9500	C52A—H52B	0.9800
C8—C9	1.392 (5)	C52A—H52C	0.9800
C8—H8	0.9500	O6A—C13A	1.188 (7)
C9—C10	1.391 (5)	C13A—C14A	1.477 (6)
C10—C11	1.396 (5)	C14A—C15A	1.3900
C10—H10	0.9500	C14A—C19A	1.3900
C11—H11	0.9500	C15A—C16A	1.3900
C12—H12A	0.9800	C15A—H15A	0.9500
C12—H12B	0.9800	C16A—C17A	1.3900
C12—H12C	0.9800	C16A—H16A	0.9500
C20—C21	1.499 (5)	C17A—C18A	1.3900
C20—H20A	0.9900	C17A—H17A	0.9500
C20—H20B	0.9900	C18A—C19A	1.3900
C21—C22	1.385 (6)	C18A—H18A	0.9500
C21—C26	1.397 (5)	C19A—H19A	0.9500
C22—C23	1.390 (6)	C29B—O52B	1.4125
C22—H22	0.9500	C29B—H29C	0.9900
C23—C24	1.372 (8)	C29B—H29D	0.9900
C23—H23	0.9500	O52B—C52B	1.5004
C24—C25	1.378 (8)	C52B—H52D	0.9800
C24—H24	0.9500	C52B—H52E	0.9800
C25—C26	1.384 (6)	C52B—H52F	0.9800
C25—H25	0.9500	O6B—C13B	1.1887
C26—H26	0.9500	C13B—C14B	1.500 (12)
C27—H27A	0.9900	C14B—C15B	1.3900
C27—H27B	0.9900	C14B—C19B	1.3900
C28—C29B	1.500 (8)	C15B—C16B	1.3900
C28—C29A	1.531 (8)	C15B—H15B	0.9500
C30—C34	1.524 (4)	C16B—C17B	1.3900
C30—H30	1.0000	C16B—H16B	0.9500
C31—C35	1.500 (4)	C17B—C18B	1.3900
C31—C32	1.521 (4)	C17B—H17B	0.9500
C31—H31	1.0000	C18B—C19B	1.3900
C32—C33	1.526 (4)	C18B—H18B	0.9500
C32—H32	1.0000	C19B—H19B	0.9500
C33—C34	1.525 (4)		
			
C6—O1—C1	118.5 (2)	C33—C34—H34	108.8
C13B—O2—C2	140.1 (3)	O11—C35—C31	106.3 (3)
C13A—O2—C2	114.2 (3)	O11—C35—H35A	110.5
C3—O3—C20	114.2 (2)	C31—C35—H35A	110.5
C30—O4—C4	116.9 (2)	O11—C35—H35B	110.5
C1—O5—C5	111.1 (2)	C31—C35—H35B	110.5
C9—O7—C12	117.8 (3)	H35A—C35—H35B	108.7
C28—O8—C27	114.6 (2)	O12—C36—O11	122.7 (5)
C30—O10—C31	114.5 (2)	O12—C36—C37	125.1 (4)
C36—O11—C35	117.6 (4)	O11—C36—C37	112.2 (5)
C33—O13—C38	113.6 (2)	C36—C37—H37A	109.5
C32—O14—C45	111.6 (2)	C36—C37—H37B	109.5
N2—N1—C34	113.9 (3)	H37A—C37—H37B	109.5
N3—N2—N1	172.4 (4)	C36—C37—H37C	109.5
O1—C1—O5	108.7 (2)	H37A—C37—H37C	109.5
O1—C1—C2	107.3 (2)	H37B—C37—H37C	109.5
O5—C1—C2	107.6 (2)	O13—C38—C39	109.5 (3)
O1—C1—H1	111.0	O13—C38—H38A	109.8
O5—C1—H1	111.0	C39—C38—H38A	109.8
C2—C1—H1	111.0	O13—C38—H38B	109.8
O2—C2—C3	107.4 (3)	C39—C38—H38B	109.8
O2—C2—C1	110.2 (3)	H38A—C38—H38B	108.2
C3—C2—C1	112.1 (2)	C44—C39—C40	118.7 (4)
O2—C2—H2	109.0	C44—C39—C38	120.6 (4)
C3—C2—H2	109.0	C40—C39—C38	120.7 (4)
C1—C2—H2	109.0	C41—C40—C39	122.1 (5)
O3—C3—C2	107.8 (2)	C41—C40—H40	118.9
O3—C3—C4	111.4 (2)	C39—C40—H40	118.9
C2—C3—C4	109.9 (2)	C40—C41—C42	118.3 (5)
O3—C3—H3	109.3	C40—C41—H41	120.8
C2—C3—H3	109.3	C42—C41—H41	120.8
C4—C3—H3	109.3	C43—C42—C41	119.6 (5)
O4—C4—C3	107.2 (2)	C43—C42—H42	120.2
O4—C4—C5	112.4 (2)	C41—C42—H42	120.2
C3—C4—C5	107.5 (2)	C42—C43—C44	121.5 (7)
O4—C4—H4	109.9	C42—C43—H43	119.2
C3—C4—H4	109.9	C44—C43—H43	119.2
C5—C4—H4	109.9	C39—C44—C43	119.7 (6)
O5—C5—C27	108.9 (2)	C39—C44—H44	120.1
O5—C5—C4	107.5 (2)	C43—C44—H44	120.1
C27—C5—C4	116.7 (2)	O14—C45—C46	109.9 (3)
O5—C5—H5	107.8	O14—C45—H45A	109.7
C27—C5—H5	107.8	C46—C45—H45A	109.7
C4—C5—H5	107.8	O14—C45—H45B	109.7
C11—C6—O1	125.0 (3)	C46—C45—H45B	109.7
C11—C6—C7	119.6 (3)	H45A—C45—H45B	108.2
O1—C6—C7	115.3 (3)	C51—C46—C47	118.6 (3)
C8—C7—C6	120.4 (3)	C51—C46—C45	121.5 (3)
C8—C7—H7	119.8	C47—C46—C45	119.8 (3)
C6—C7—H7	119.8	C48—C47—C46	120.9 (4)
C7—C8—C9	120.2 (3)	C48—C47—H47	119.5
C7—C8—H8	119.9	C46—C47—H47	119.5
C9—C8—H8	119.9	C49—C48—C47	120.3 (4)
O7—C9—C10	125.1 (3)	C49—C48—H48	119.9
O7—C9—C8	115.6 (3)	C47—C48—H48	119.9
C10—C9—C8	119.3 (3)	C48—C49—C50	120.3 (4)
C9—C10—C11	120.3 (3)	C48—C49—H49	119.8
C9—C10—H10	119.9	C50—C49—H49	119.8
C11—C10—H10	119.9	C49—C50—C51	119.1 (4)
C6—C11—C10	120.1 (3)	C49—C50—H50	120.5
C6—C11—H11	119.9	C51—C50—H50	120.5
C10—C11—H11	119.9	C46—C51—C50	120.8 (4)
O7—C12—H12A	109.5	C46—C51—H51	119.6
O7—C12—H12B	109.5	C50—C51—H51	119.6
H12A—C12—H12B	109.5	O52A—C29A—C28	114.0 (5)
O7—C12—H12C	109.5	O52A—C29A—H29A	108.7
H12A—C12—H12C	109.5	C28—C29A—H29A	108.7
H12B—C12—H12C	109.5	O52A—C29A—H29B	108.7
O3—C20—C21	109.0 (3)	C28—C29A—H29B	108.7
O3—C20—H20A	109.9	H29A—C29A—H29B	107.6
C21—C20—H20A	109.9	C29A—O52A—C52A	113.0 (7)
O3—C20—H20B	109.9	O52A—C52A—H52A	109.5
C21—C20—H20B	109.9	O52A—C52A—H52B	109.5
H20A—C20—H20B	108.3	H52A—C52A—H52B	109.5
C22—C21—C26	118.4 (4)	O52A—C52A—H52C	109.5
C22—C21—C20	120.8 (3)	H52A—C52A—H52C	109.5
C26—C21—C20	120.8 (4)	H52B—C52A—H52C	109.5
C21—C22—C23	120.9 (4)	O6A—C13A—O2	125.6 (5)
C21—C22—H22	119.6	O6A—C13A—C14A	124.5 (5)
C23—C22—H22	119.6	O2—C13A—C14A	109.9 (4)
C24—C23—C22	120.0 (5)	C15A—C14A—C19A	120.0
C24—C23—H23	120.0	C15A—C14A—C13A	117.7 (3)
C22—C23—H23	120.0	C19A—C14A—C13A	122.3 (3)
C23—C24—C25	119.9 (4)	C14A—C15A—C16A	120.0
C23—C24—H24	120.1	C14A—C15A—H15A	120.0
C25—C24—H24	120.1	C16A—C15A—H15A	120.0
C24—C25—C26	120.5 (4)	C17A—C16A—C15A	120.0
C24—C25—H25	119.7	C17A—C16A—H16A	120.0
C26—C25—H25	119.7	C15A—C16A—H16A	120.0
C25—C26—C21	120.2 (4)	C18A—C17A—C16A	120.0
C25—C26—H26	119.9	C18A—C17A—H17A	120.0
C21—C26—H26	119.9	C16A—C17A—H17A	120.0
O8—C27—C5	109.5 (2)	C17A—C18A—C19A	120.0
O8—C27—H27A	109.8	C17A—C18A—H18A	120.0
C5—C27—H27A	109.8	C19A—C18A—H18A	120.0
O8—C27—H27B	109.8	C18A—C19A—C14A	120.0
C5—C27—H27B	109.8	C18A—C19A—H19A	120.0
H27A—C27—H27B	108.2	C14A—C19A—H19A	120.0
O9—C28—O8	124.6 (3)	O52B—C29B—C28	104.8 (3)
O9—C28—C29B	123.5 (3)	O52B—C29B—H29C	110.8
O8—C28—C29B	110.6 (3)	C28—C29B—H29C	110.8
O9—C28—C29A	125.1 (4)	O52B—C29B—H29D	110.8
O8—C28—C29A	110.2 (4)	C28—C29B—H29D	110.8
O4—C30—O10	111.7 (2)	H29C—C29B—H29D	108.9
O4—C30—C34	110.0 (2)	C29B—O52B—C52B	113.5
O10—C30—C34	108.5 (2)	O52B—C52B—H52D	109.5
O4—C30—H30	108.9	O52B—C52B—H52E	109.5
O10—C30—H30	108.9	H52D—C52B—H52E	109.5
C34—C30—H30	108.9	O52B—C52B—H52F	109.5
O10—C31—C35	106.2 (2)	H52D—C52B—H52F	109.5
O10—C31—C32	110.0 (2)	H52E—C52B—H52F	109.5
C35—C31—C32	113.1 (3)	O2—C13B—O6B	111.7 (2)
O10—C31—H31	109.1	O2—C13B—C14B	122.5 (7)
C35—C31—H31	109.1	O6B—C13B—C14B	125.3 (6)
C32—C31—H31	109.1	C15B—C14B—C19B	120.0
O14—C32—C31	108.2 (2)	C15B—C14B—C13B	119.4 (10)
O14—C32—C33	110.6 (2)	C19B—C14B—C13B	120.5 (10)
C31—C32—C33	109.6 (2)	C16B—C15B—C14B	120.0
O14—C32—H32	109.5	C16B—C15B—H15B	120.0
C31—C32—H32	109.5	C14B—C15B—H15B	120.0
C33—C32—H32	109.5	C15B—C16B—C17B	120.0
O13—C33—C34	108.7 (3)	C15B—C16B—H16B	120.0
O13—C33—C32	111.4 (2)	C17B—C16B—H16B	120.0
C34—C33—C32	107.8 (3)	C16B—C17B—C18B	120.0
O13—C33—H33	109.6	C16B—C17B—H17B	120.0
C34—C33—H33	109.6	C18B—C17B—H17B	120.0
C32—C33—H33	109.6	C17B—C18B—C19B	120.0
N1—C34—C30	108.4 (3)	C17B—C18B—H18B	120.0
N1—C34—C33	111.8 (3)	C19B—C18B—H18B	120.0
C30—C34—C33	110.2 (2)	C18B—C19B—C14B	120.0
N1—C34—H34	108.8	C18B—C19B—H19B	120.0
C30—C34—H34	108.8	C14B—C19B—H19B	120.0
			
C6—O1—C1—O5	−77.2 (3)	C31—C32—C33—C34	56.9 (3)
C6—O1—C1—C2	166.6 (2)	N2—N1—C34—C30	149.6 (3)
C5—O5—C1—O1	178.8 (2)	N2—N1—C34—C33	−88.8 (3)
C5—O5—C1—C2	−65.3 (3)	O4—C30—C34—N1	59.5 (3)
C13B—O2—C2—C3	−140.2 (4)	O10—C30—C34—N1	−178.0 (2)
C13A—O2—C2—C3	−113.4 (4)	O4—C30—C34—C33	−63.1 (3)
C13B—O2—C2—C1	97.5 (5)	O10—C30—C34—C33	59.4 (3)
C13A—O2—C2—C1	124.2 (3)	O13—C33—C34—N1	59.6 (3)
O1—C1—C2—O2	−67.7 (3)	C32—C33—C34—N1	−179.5 (2)
O5—C1—C2—O2	175.5 (2)	O13—C33—C34—C30	−179.8 (2)
O1—C1—C2—C3	172.8 (2)	C32—C33—C34—C30	−58.9 (3)
O5—C1—C2—C3	55.9 (3)	C36—O11—C35—C31	−166.5 (3)
C20—O3—C3—C2	−128.0 (3)	O10—C31—C35—O11	74.3 (3)
C20—O3—C3—C4	111.4 (3)	C32—C31—C35—O11	−164.9 (3)
O2—C2—C3—O3	64.8 (3)	C35—O11—C36—O12	0.7 (6)
C1—C2—C3—O3	−174.0 (2)	C35—O11—C36—C37	−179.2 (3)
O2—C2—C3—C4	−173.7 (2)	C33—O13—C38—C39	178.9 (3)
C1—C2—C3—C4	−52.5 (3)	O13—C38—C39—C44	−54.9 (5)
C30—O4—C4—C3	143.2 (2)	O13—C38—C39—C40	128.7 (4)
C30—O4—C4—C5	−98.8 (3)	C44—C39—C40—C41	1.9 (8)
O3—C3—C4—O4	−65.2 (3)	C38—C39—C40—C41	178.4 (4)
C2—C3—C4—O4	175.5 (2)	C39—C40—C41—C42	−1.4 (8)
O3—C3—C4—C5	173.7 (2)	C40—C41—C42—C43	1.2 (9)
C2—C3—C4—C5	54.3 (3)	C41—C42—C43—C44	−1.7 (11)
C1—O5—C5—C27	−162.9 (2)	C40—C39—C44—C43	−2.3 (9)
C1—O5—C5—C4	69.9 (3)	C38—C39—C44—C43	−178.8 (5)
O4—C4—C5—O5	−179.9 (2)	C42—C43—C44—C39	2.2 (10)
C3—C4—C5—O5	−62.1 (3)	C32—O14—C45—C46	174.7 (3)
O4—C4—C5—C27	57.5 (3)	O14—C45—C46—C51	72.3 (4)
C3—C4—C5—C27	175.4 (2)	O14—C45—C46—C47	−111.5 (4)
C1—O1—C6—C11	36.4 (4)	C51—C46—C47—C48	−1.0 (7)
C1—O1—C6—C7	−148.5 (3)	C45—C46—C47—C48	−177.3 (4)
C11—C6—C7—C8	1.8 (5)	C46—C47—C48—C49	0.3 (9)
O1—C6—C7—C8	−173.6 (3)	C47—C48—C49—C50	1.1 (9)
C6—C7—C8—C9	1.2 (5)	C48—C49—C50—C51	−1.8 (7)
C12—O7—C9—C10	2.3 (5)	C47—C46—C51—C50	0.3 (6)
C12—O7—C9—C8	−176.2 (3)	C45—C46—C51—C50	176.6 (3)
C7—C8—C9—O7	174.5 (3)	C49—C50—C51—C46	1.1 (6)
C7—C8—C9—C10	−4.2 (5)	O9—C28—C29A—O52A	12.9 (14)
O7—C9—C10—C11	−174.4 (3)	O8—C28—C29A—O52A	−163.8 (7)
C8—C9—C10—C11	4.1 (5)	C29B—C28—C29A—O52A	101.6 (15)
O1—C6—C11—C10	173.1 (3)	C28—C29A—O52A—C52A	76.1 (11)
C7—C6—C11—C10	−1.8 (5)	C13B—O2—C13A—O6A	139.8 (9)
C9—C10—C11—C6	−1.2 (5)	C2—O2—C13A—O6A	−8.3 (8)
C3—O3—C20—C21	173.9 (3)	C13B—O2—C13A—C14A	−40.9 (3)
O3—C20—C21—C22	127.3 (4)	C2—O2—C13A—C14A	171.0 (3)
O3—C20—C21—C26	−52.2 (4)	O6A—C13A—C14A—C15A	13.2 (7)
C26—C21—C22—C23	0.0 (6)	O2—C13A—C14A—C15A	−166.1 (3)
C20—C21—C22—C23	−179.6 (4)	O6A—C13A—C14A—C19A	−165.3 (5)
C21—C22—C23—C24	1.0 (7)	O2—C13A—C14A—C19A	15.3 (5)
C22—C23—C24—C25	−1.0 (8)	C19A—C14A—C15A—C16A	0.0
C23—C24—C25—C26	−0.1 (8)	C13A—C14A—C15A—C16A	−178.6 (3)
C24—C25—C26—C21	1.1 (7)	C14A—C15A—C16A—C17A	0.0
C22—C21—C26—C25	−1.0 (6)	C15A—C16A—C17A—C18A	0.0
C20—C21—C26—C25	178.6 (4)	C16A—C17A—C18A—C19A	0.0
C28—O8—C27—C5	−167.9 (3)	C17A—C18A—C19A—C14A	0.0
O5—C5—C27—O8	−74.5 (3)	C15A—C14A—C19A—C18A	0.0
C4—C5—C27—O8	47.3 (3)	C13A—C14A—C19A—C18A	178.5 (3)
C27—O8—C28—O9	−7.2 (5)	O9—C28—C29B—O52B	116.4 (4)
C27—O8—C28—C29B	−174.6 (4)	O8—C28—C29B—O52B	−76.0 (3)
C27—O8—C28—C29A	169.5 (6)	C29A—C28—C29B—O52B	14.9 (11)
C4—O4—C30—O10	105.5 (3)	C28—C29B—O52B—C52B	−75.4 (2)
C4—O4—C30—C34	−134.0 (3)	C13A—O2—C13B—O6B	−40.8 (5)
C31—O10—C30—O4	61.5 (3)	C2—O2—C13B—O6B	7.8 (5)
C31—O10—C30—C34	−59.9 (3)	C13A—O2—C13B—C14B	131.9 (10)
C30—O10—C31—C35	−178.1 (3)	C2—O2—C13B—C14B	−179.4 (9)
C30—O10—C31—C32	59.1 (3)	O2—C13B—C14B—C15B	−172.3 (7)
C45—O14—C32—C31	−137.5 (3)	O6B—C13B—C14B—C15B	−0.6 (12)
C45—O14—C32—C33	102.4 (3)	O2—C13B—C14B—C19B	6.9 (15)
O10—C31—C32—O14	−176.8 (2)	O6B—C13B—C14B—C19B	178.6 (7)
C35—C31—C32—O14	64.6 (3)	C19B—C14B—C15B—C16B	0.0
O10—C31—C32—C33	−56.1 (3)	C13B—C14B—C15B—C16B	179.2 (14)
C35—C31—C32—C33	−174.7 (3)	C14B—C15B—C16B—C17B	0.0
C38—O13—C33—C34	−135.5 (3)	C15B—C16B—C17B—C18B	0.0
C38—O13—C33—C32	105.8 (3)	C16B—C17B—C18B—C19B	0.0
O14—C32—C33—O13	−64.6 (3)	C17B—C18B—C19B—C14B	0.0
C31—C32—C33—O13	176.2 (2)	C15B—C14B—C19B—C18B	0.0
O14—C32—C33—C34	176.2 (2)	C13B—C14B—C19B—C18B	−179.2 (14)

### (RSTN) 4-Methoxyphenyl 4-O-[6-O-acetyl-2-azido-3,4-O-benzyl-2-deoxy-α-D-glucopyranosyl]-2-O-benzoyl-3-O-benzyl-6-O-methoxyoacetyl-β-D-glucopyranoside Hydrogen-bond geometry (Å, º)

Cg3, Cg5 and Cg6 are the centroids of the C6–C11, C21–C26 and C39–C44 phenyl rings, respectively.

**Table:**

*D*—H···*A*	*D*—H	H···*A*	*D*···*A*	*D*—H···*A*
C5—H5···O1^i^	1.00	2.46	3.439 (4)	168
C27—H27*B*···O11	0.99	2.55	3.499 (4)	161
C44—H44···N3	0.95	2.64	3.564 (5)	
C45—H45*A*···O12^ii^	0.99	2.51	3.488 (5)	168
C47—H47···O52*A*^iii^	0.95	2.59	3.269 (4)	128
C48—H48···O9^iii^	0.95	2.65	3.569 (6)	164
C3—H3···*Cg*3^i^	1.00	2.96	3.915 (3)	161
C4—H4···*Cg*3^iv^	1.00	2.96	3.920 (3)	161
C12—H12*B*···*Cg*5^i^	0.98	2.71	3.563 (3)	145
C16*A*—H16*A*···*Cg*6^v^	0.95	2.88	3.713 (3)	147
C25—H25···*Cg*5^v^	0.95	2.94	3.717 (4)	140

Symmetry codes: (i) −*x*+1/2, *y*+1/2, −*z*; (ii) −*x*+1, *y*, −*z*; (iii) −*x*+1, *y*+1, −*z*; (iv) −*x*+1/2, *y*−1/2, −*z*; (v) −*x*+1/2, *y*−1/2, −*z*+1.
